# A linear pathway for inositol pyrophosphate metabolism revealed by ^18^O labeling and model reduction

**DOI:** 10.1371/journal.pcbi.1013680

**Published:** 2025-11-10

**Authors:** Jacques Hermes, Geun-Don Kim, Guizhen Liu, Maria Giovanna De Leo, Andreas Mayer, Henning Jessen, Jens Timmer

**Affiliations:** 1 Institute of Physics, University of Freiburg, Freiburg, Germany; 2 Freiburg Center for Data Analysis and Modelling (FDM), University of Freiburg, Freiburg, Germany; 3 Centre for Integrative Biological Signalling Studies (CIBSS), University of Freiburg, Freiburg, Germany; 4 Département d’immunobiologie, Université de Lausanne, Epalinges, Switzerland; 5 Institute of Organic Chemistry, University of Freiburg, Freiburg, Germany; Clemson University, UNITED STATES OF AMERICA

## Abstract

The homeostasis of intracellular inorganic phosphate is essential for eukaryotic metabolism and is regulated by the INPHORS signalling pathway, which employs inositol pyrophosphates (IPPs) as key intermediary messengers. This study investigates the metabolic pathways of inositol pyrophosphates (IPPs) in the yeast cell line Pho^ΔSPX^ and the human tumor cell line HCT116. Utilizing pulse-labelling experiments with ^18^O water and ordinary differential equation (ODE) models, we explore the synthesis and turnover of the highly phosphorylated IPP, 1,5-InsP_8_. Our findings challenge the notion that 1,5-InsP_8_ can be synthesized through distinct routes, revealing a linear reaction sequence in both systems. Employing model reduction via the profile likelihood method, we achieved statistically concise identifiability analysis that led to significant biological insights. In yeast, we determined that 1,5-InsP_8_ production primarily occurs through the phosphorylation of 5-InsP_7_, with the pathway involving 1-InsP_7_ deemed unnecessary as its removal did not compromise model accuracy. Crucially, this prediction of altered IPP concentrations was validated experimentally in *vip1*Δ and *kcs1*Δ knockout strains, providing orthogonal biological support for the reduced model. In HCT116 cells, 1,5-InsP_8_ synthesis is mainly driven by 1-InsP_7_, with variations observed across different experimental conditions. These results underscore the utility of model reduction in enhancing our understanding of metabolic pathways, coupling predictive modeling with experimental validation, and providing a framework for future investigations into the regulation and implications of linear IPP pathways in eukaryotic cells.

## Author summary

This research reveals how cells produce 1,5-InsP_8_, a key molecule in phosphate homeostasis, a vital part of cellular metabolism. Using isotope tracing and mathematical modelling, the study shows that both yeast and human cancer cells follow a linear, rather than branching, pathway to build this molecule. The analysis identified essential steps in the process, challenging previous assumptions about how these molecules are synthesized. By improving our understanding of inositol pyrophosphate metabolism, this work offers new insights into how cells regulate phosphate levels, with implications for cellular energy supply and metabolism in general.

## Introduction

All living organisms utilize signalling molecules to respond to external stimuli and maintain internal stability. Inositol pyrophosphates (IPPs) are a class of signalling molecules that is evolutionarily conserved in eukaryotic cells [1]. IPPs are generated as part of the INPHORS (intracellular phosphate reception and signalling) pathway (Ref [17]). They bind to a familiy of conserved SPX domains, change their conformation and/or association state, and thereby regulate a network of SPX-carrying target proteins [2–7]. These target proteins have in many cases a direct role in phosphate transport or utilisation. Thereby, INPHORS signalling is a key regulator of intracellular phosphate homeostasis in eukaryotic cells [8–10].

IPPs occurring in cells contain one or two pyrophosphate groups attached to the inositol ring. The number and positioning of the pyrophosphate groups may confer different physiological roles [11–13]. IPPs are synthesized by several kinases using InsP_6_ as a backbone. Inositol hexakisphosphate kinases (IP6Ks in mammals; Kcs1 in yeast) add a phosphate group at the C5 position of the inositol ring, generating 5-InsP_7_ from InsP_6_ or 1,5-InsP_8_ from 1-InsP_7_ [14,15]. Diphosphoinositol pentakisphosphate kinases (PPIP5Ks in mammals; Vip1 in yeast) phosphylate at the C1 position of the inositol ring and synthesize 1-InsP_7_ from InsP_6_ or 1,5-InsP_8_ from 5-InsP_7_ [16,17]. Further IPPs have been identified in plants and mammalian cells [18,19], but at present it is not clear what physiological roles they might play. Highly phosphorylated IPPs are converted back to InsP_6_ by several phosphatases. PPIP5Ks are bidirectional enzymes containing both kinase and phosphatase activities [20,21]. Their phosphatase domain can remove the *β*-phosphate at the C1 position from 1-InsP_7_ or 1,5-InsP_8_. In mammals, diphosphoinositol polyphosphate phosphohydrolases (DIPPs) nonspecifically remove the *β*-phosphate group, leading to the conversion of IPPs back to InsP_6_ [22]. In yeast, several different phosphatases are involved in the dephosphorylation of IPPs. Ddp1, a yeast homolog of mammalian DIPPs, preferentially removes the *β*-phosphate group at the C1 position, generating 5-InsP_7_ from 1,5-InsP_8_ or InsP_6_ from 1-InsP_7_ [23–25]. Meanwhile, Siw14 removes the *β*-phosphate at the C5 position to create 1-InsP_7_ from 1,5-InsP_8_ or InsP_6_ from 5-InsP_7_ [26–28].

The levels of IPPs change quantitatively and qualitatively in response to various cellular signals and environmental stimuli. For example, when yeast cells are starved for inorganic phosphate (${\text{P}}_{\text{i}}$), the amount of overall IPPs decrease rapidly, with 1,5-InsP_8_ showing a faster and more pronounced decline than 1-InsP_7_ and 5-InsP_7_ [29]. The decrease in 1,5-InsP_8_ triggers the phosphate-responsive signal transduction (PHO) pathway. This pathway induces transcription of genes that increase the capacity for scavenging ${\text{P}}_{\text{i}}$ from the surrounding environment, and for liberating ${\text{P}}_{\text{i}}$ from polyphosphates, nucleic acids, or lipids to enable internal ${\text{P}}_{\text{i}}$ recycling [10]. 1,5-InsP_8_ responds similarly to ${\text{P}}_{\text{i}}$ starvation in mammalian cells and in plants, suggesting that its role in ${\text{P}}_{\text{i}}$ signalling is conserved [30–32]. The amount of IPPs also changes during the cell cycle, perhaps as a consequence of fluctuating ${\text{P}}_{\text{i}}$ utilization [33–36]. Changes of IPPs in turn affect the cell cycle progression, e.g., by controlling kinetochore formation [37]. In addition, environmental stresses such as hyperosmotic stress [38], oxidative stress [39,40], and heat stress [41] can affect IPPs levels.

1,5-InsP_8_ can be produced through two separate routes from InsP_6_, via 5-InsP_7_ or 1-InsP_7_. It was proposed that the main route for synthesizing 1,5-InsP_8_ from InsP_6_ is through 5-InsP_7_, while the conversion of InsP_6_ to 1-InsP_7_ by PPIP5Ks is not efficient *in vivo* [42]. This model was derived from several lines of evidence: 1) The cellular concentration of InsP_6_ is markedly higher than that of other IPPs, but its turnover rate is significantly slower compared to that of other IPPs [43], implying that a substantial amount of InsP_6_ is compartmentalized or remains unreactive. 2) Several studies showed that PPIP5Ks prefer 5-InsP_7_ as a substrate over InsP_6_ [44,45]. 3) The concentration of 1-InsP_7_ within the cell is very low, almost to the point of being undetectable under normal conditions [29,46]. While substrate preferences of the IPP enzymes could be dissected through *in vitro* studies [44,45], such information does not automatically predict the metabolite fluxes *in vivo*. Therefore, it is required to analyse IPPs synthesis *in vivo* to understand the 1,5-InsP_8_ synthesis pathway. Here we used data from pulse-labelling experiments with ^18^O water to dissect the kinetics of IPP turnover and ordinary differential equation (ODE) models to reveal the relevant metabolite fluxes. In order to estimate the parameters of our ODE models, we used the modelling tool box *dMod* [47], which was developed in our group, to fit the ODE model to the pulse-labelling data. Furthermore we applied a method called profile likelihood [48,49] to perform a statistical analysis of the estimated parameters’ uncertainties. Based on this we reparameterized our models, i.e. adjusted the model structures, in a process called model reduction [50,51]. Our work was designed to test wether both the 1-InsP_7_ and 5-InsP_7_ branches are required to reproduce the labeling dynamics. If one branch can be consistently eliminated during model reduction without loss of fit, then a linear topology provides the most parsimonious explanation consistent with the data. Using this approach, we found that the analysed organisms, the yeast *Saccharomyces cerevisiae* and a human tumor cell-line (HCT116), synthesize 1,5-InsP_8_ through distinct routes. In both cases however, the statistically preferred version of their IPP metabolic routes is not a cycle but rather a linear reaction network.

## Results

**Fig 1:**
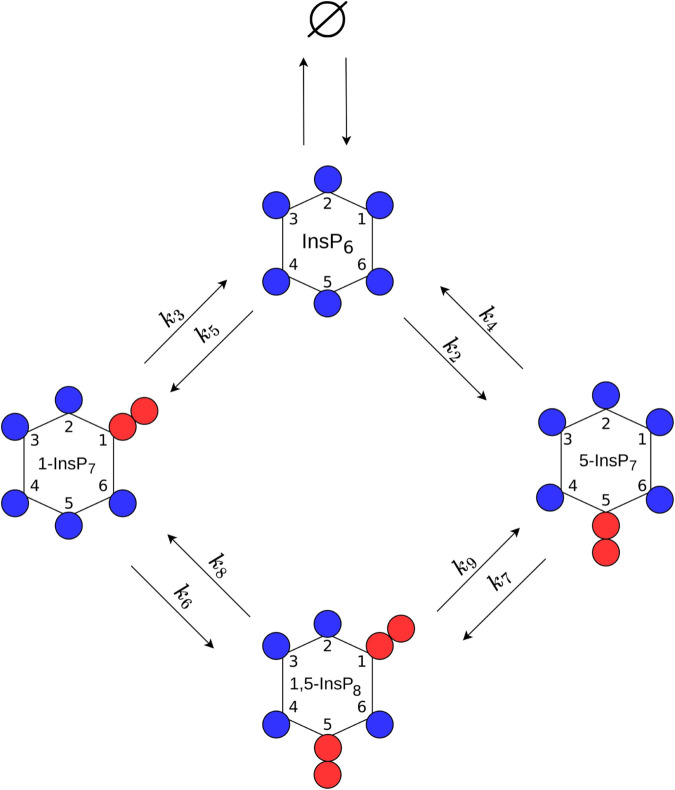
Scheme of the cyclic production pathway of 1,5-InsP_8_ upon which the “full” ODE-model is based.

**Fig 2:**
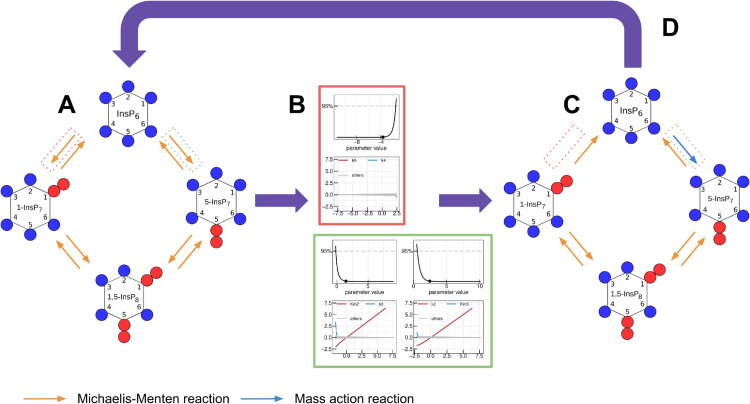
Schematic overview of the model reduction procedure. (A) Full model fitted to the experimental data. All reactions are initially represented as Michaelis-Menten rate laws (orange arrows, see legend at bottom). Two reactions are highlighted: one enclosed in a red dotted box, one in a green dotted box. (B) Profile likelihoods calculated to assess parameter identifiability. The reaction in the red box is identified as non-contributing, while the parameters in the green box are identified as coupled. (C) Reduced model after this step. The reaction in the red box has been removed, while the reaction in the green box has been simplified to a mass-action process (blue arrow). (D) Iterative procedure. The reduced model is refitted, and steps (A–C) are repeated until all parameters are identifiable and a parsimonious model structure is obtained.

In this work we analysed two different biological organisms, baker’s yeast and HCT116. The data sets were first analysed using the same initial, in the following called “full”, mathematical model (Fig 1). The data-sets analysed in this manuscript stem from a recently published approach, in which cellular IPs are rapidly pulse-labelled through ^18^O water [52,53]. All experiments contain three biological replicates per time point. Since it is expected that the metabolic cycle has different kinetics between both organisms, the model was fitted and reduced for both organisms independently. However, the applied methods and strategies were identical in all analyses to ensure statistically sound results. For every iteration a multi-start fit approach was chosen, i.e. multiple fits with individual and randomly sampled initial parameter vectors were performed. To obtain statistically sound and comparable results, 1000 multi-starts were performed for each iteration. A schematic overview of the reduction procedure is given in Fig 2. The process begins by fitting the full model to the experimental data (A). Profile likelihood analysis is then performed to assess parameter identifiability (B). Non-identifiable parameters are reduced either by structurally modifying the underlying rate equation or by removing the corresponding reactions (C). The resulting reduced model is subsequently refitted, and the procedure is iterated until all parameters are identifiable and a parsimonious model structure is obtained (D). In the following, the model reduction steps leading to the final fully reduced model are described for both organisms independently. The mathematical modelling techniques employed in this work are explained in detail in the Materials and Methods section.

### Yeast

**Fig 3:**
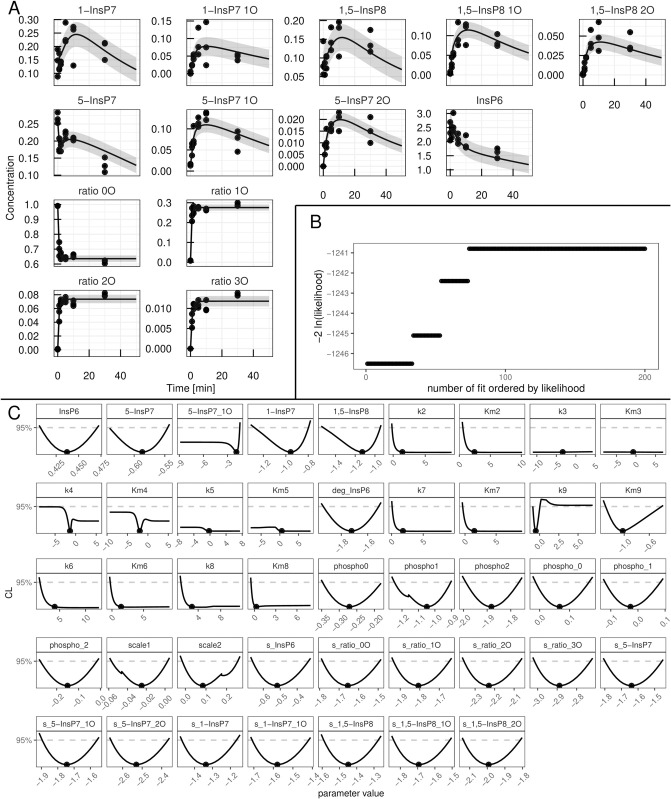
Analysis of the yeast data-set with the “full” mathematical model. (A) Fit of the mathematical model with the best-fit parameters (line) to the experimental data (points). The values are given in *μ*M except for the four species in the lower left of the panel which represent ratios of 1,2,3-labelled to 0-unlabelled ATP *γ*-phosphate. (B) Likelihood values of the 200 best multi-start fits, ordered by lowest likelihood value. (C) Profiles of the best-fit parameters of the full model (line). Best-fit parameter value are represented as points. Parameter values (x-axis) are displayed on log10 scale.

**Fig 4:**
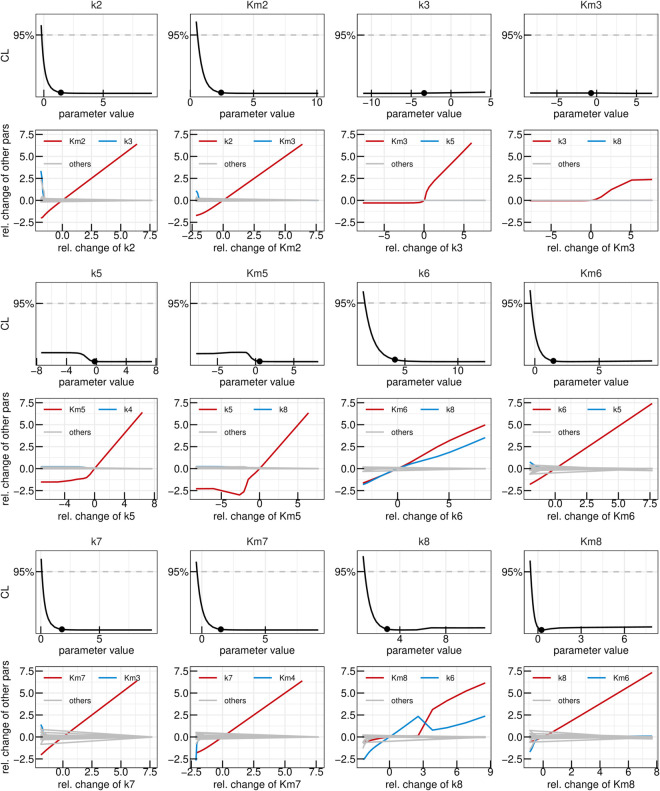
First reduction step in the yeast model. Profiles of the parameters considered for the first model reduction step in the yeast metabolic cycle. Best-fit parameter value are represented as points. Parameter values (x-axis) are displayed on log10 scale. Below each profile, the dependencies of all other parameters on the profiled parameter are plotted. Coupling strength is quantified by the relative change of a secondary parameter from its best-fit value after re-optimizing all parameters at each fixed value of the profiled parameter (x-axis). Both axes are shown on a log_10_ scale. The strongest dependency is highlighted in red, the next strongest in blue, and the remaining dependencies in gray.

In the yeast dataset, the levels of 1-InsP_7_ were not shown because of their low abundancein the samples [52]. For that reason, we reinvestigated the ^18^O labelling of IPPs under the high ${\text{P}}_{\text{i}}$ conditions in the cytosol, which favours the accumulation of all IPP species [29]. To this end, we overexpressed the truncated form of the low-affinity phosphate transporter Pho90 (Pho90^ΔSPX^), which lacks the restraining SPX domain and allows enhanced ${\text{P}}_{\text{i}}$ import into the cells [54]. The cells were first grown in low phosphate medium (0.2 mM) and then transferred to high phosphate medium (50 mM) with 50% ^18^O water to generate ${\text{P}}_{\text{i}}$ influx and enhanced IPP synthesis. For further details on the generation of the data set, we refer to the Materials and Methods section. In a first step, the yeast data-set was analysed using the “full” ODE model, which yielded a set of best-fit parameters which adequately described the dynamics observed in the data and yielded a Bayesian information criterion value (BIC) of -1011,9. The data as well as the time-courses obtained via the mathematical model with best-fit parameters are shown in panel A of Fig 3. In panel B, the likelihood values of the 200 best fits out of the 1000 multi-starts are shown sorted by the lowest likelihood. Such a plot, called waterfall plot, indicates that the best fit found in the analysis is found by eight different initial parameter configurations, providing evidence that the found optimum is in fact a global and not just a local one [48]. In a next step, parameter profiles were calculated, as shown in Fig 3C. This resulted in six model reactions where the rate parameters *k*_*i*_ and the Michaelis-Menten (MM) constant $K_{M_{i}}$ are both not identifiable, namely i∈{2,3,5,6,7,8}. A deviation from the profiled parameters best-fit value can thus be compensated by the re-optimization of other parameter values resulting in the same likelihood values, therefore being called coupled to or dependent on the compensating parameters. The dependencies of the non-identifiable *k*_*i*_s and $K_{M_{i}}$s are graphically displayed in Fig 4 where the two strongest dependencies are displayed as paths in red and blue respectively and the dependencies of the remaining model parameters are shown in gray. In most panels, only the strongest dependency in red is visible since that parameter only strongly depends on one other parameter and the gray lines are thus overlapping the blue line. The parameter profile of interest is again plotted above its respective paths. Except for *k*_6_ and *k*_8_, the parameters solely depend on the *K*_*M*_ or *k* values of their respective reactions. Furthermore, if the profiled parameter value is increased the respective counterpart also increases in order to compensate for the change. Combining this observation with the fact that the *K*_*M*_ values of interest have profiles with confidence intervals open to +∞ we see they can take very large values without worsening the likelihood. This supports reducing these reactions to mass-action reactions. Such mass-action reactions occur in a regime of Michaelis-Menten (MM) reactions where the *K*_*M*_ value is much larger than the available substrate concentration [55]. For *k*_6_ and *k*_8_ this reduction is also valid, since their *K*_*M*_ values, which are the parameters that are being reduced, only depend on their respective *k* values. However, we do not expect *k*_6_ and *k*_8_ to become identifiable via this step, due to their strong dependencies upon each other, as seen in Fig 4.

**Fig 5:**
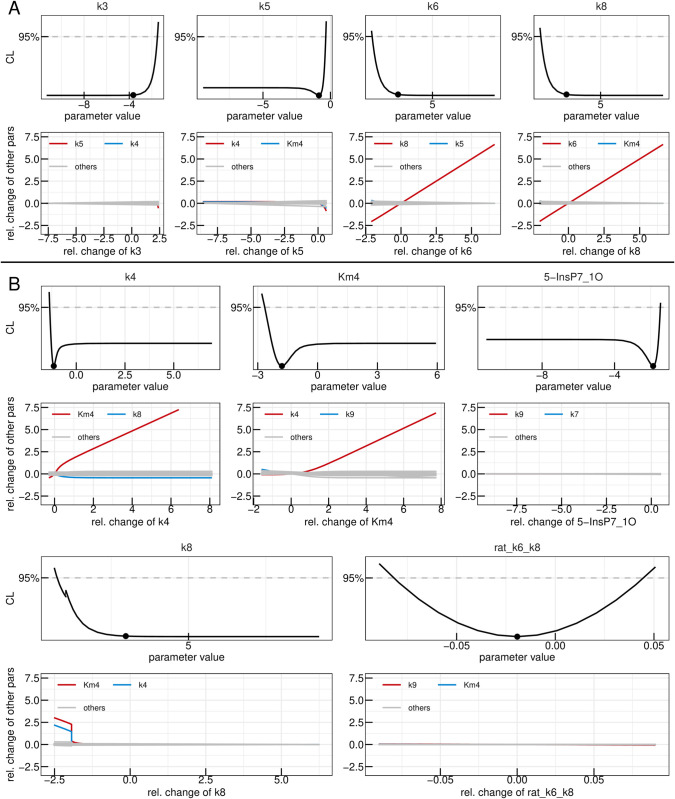
Second and third reduction steps in the yeast model. Profiles of the parameters considered for the second (A) and third (B) model reduction step in the yeast metabolic cycle. Best-fit parameter value are represented as points. Parameter values (x-axis) are displayed on log10 scale. Below each profile, the dependencies of all other parameters on the profiled parameter are plotted. Coupling strength is quantified by the relative change of a secondary parameter from its best-fit value after re-optimizing all parameters at each fixed value of the profiled parameter (x-axis). Both axes are shown on a log_10_ scale. The strongest dependency is highlighted in red, the next strongest in blue, and the remaining dependencies in gray.

A subsequent multi-start fit of the so reduced model to the same data set showed no visible changes in goodness-of-fit, while BIC improved to -1045,2. Examining the parameter profiles resulting from this refitting implied that also the reaction rates *k*_3_ and *k*_5_ could be set to zero, since their profiles are open towards -∞ on the log scale (or zero on the linear scale), as shown in Fig 5A. Furthermore, no significant dependencies for small values of these parameters are present, demonstrating that for values smaller then the best-fit one, other parameters do not need to be adjusted in order to keep the best likelihood value, justifying setting these reaction rates to zero on linear scale. The profiles of both *k*_8_ and *k*_6_ on the other hand are still open towards +∞ on the log scale, meaning both reactions could be set to arbitrarily high values without significantly changing the model’s capacity of describing the data. The remaining dependencies of the two parameters in Fig 5A indicate that they linearly dependent upon one-another signifying that one can introduce the parameter transformation $k_{6}=\frac{k_{6}}{k_{8}}\;\cdot \;k_{8}={\text{ratio}}_{k_{6},k_{8}}\;\cdot \;k_{8}$. It is expected, that the ratio between the two rates becomes identifiable.

**Fig 6:**
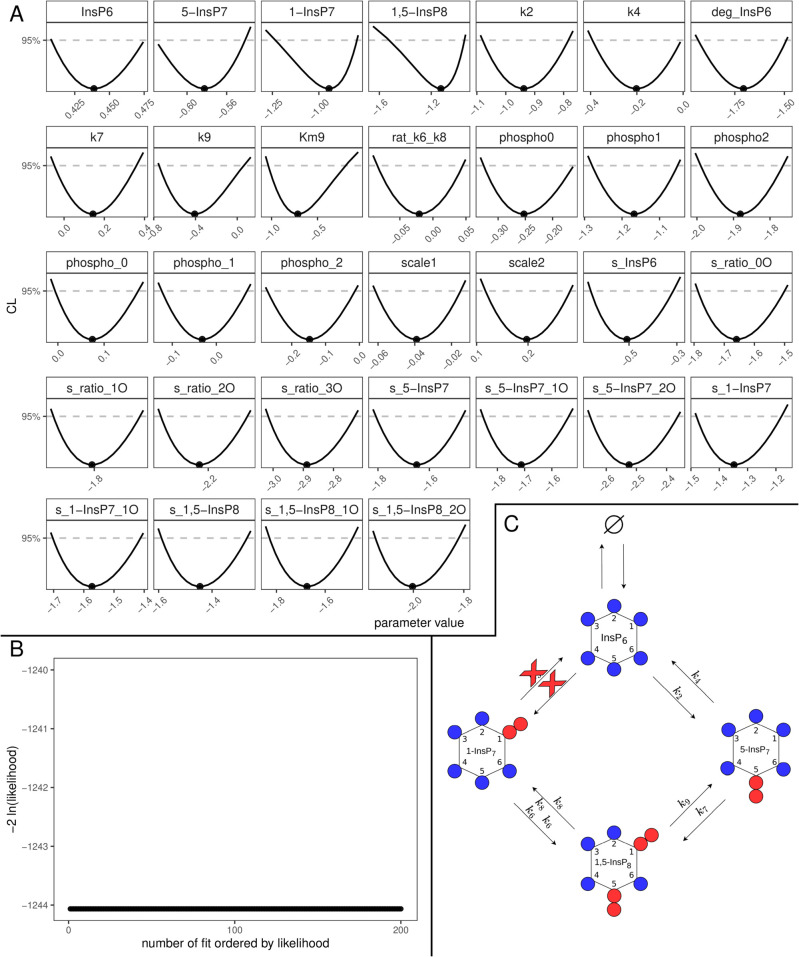
Fully reduced yeast model. (A) Profiles of the best-fit parameters of the reduced model (line). Best-fit parameter value are represented as points. Parameter values (x-axis) are displayed on log10 scale. (B) Likelihood values of the 200 best multi-start fits, ordered by lowest likelihood value. (C) Representation of the statistically favored transition scheme, displaying a chain-like pattern rather than a cycle.

The now twice reduced model was once again fitted to the data, yielding an unchanged description of the data while resulting in a BIC of -1055,9. In a last reduction step, the reaction involving *k*_4_ and $K_{M_{4}}$ are reduced to mass action, following the same reasoning as for the other mass-action reduction steps. Additionally, the initial value for single labelled 5-InsP_7_ was also reduced to 0 following the reasoning of the previous reductions of *k*_3_ and *k*_5_. The profiles and dependencies of these three parameters are shown in Fig 5B, together with the identifiable profile of the ratio between *k*_6_ and *k*_8_ and the profile of *k*_8_ which, as expected, is still open towards +∞. Since the goodness-of-fit is not limited by large values of *k*_8_ its value was fixed to 10^5^ on the linear scale. The final, completely reduced model yielded profiles shown in Fig 6A and a BIC of -1069.5. It is completely identifiable as seen in the parabolic shapes of every single calculated profile. Note, that only the description of the reaction involving *k*_9_ requires the use of Michaelis-Menten kinetic since both *k*_9_ and $K_{M_{9}}$ are identifiable parameters. In panel B of Fig 6 the waterfall plot of the 200 best multi-starts is plotted, where no step can be observed since all 200 fits ended up at the same best likelihood value, which indicates high trust in the employed numerical optimisations. To further strengthen the confidence in the obtained optimum, the distribution for the parameters of the 200 best runs is displayed in S7 Fig which shows a point like distribution for the parameters across the selected fits. Furthermore a time vs residual plot is shown in Fig S6 Fig providing no evidence of systematic bias of the residuals over time thus indicating that the fits are not unduly driven by individual noisy points.

Finally, in panel C, a scheme of the statistically favoured version of the reaction pathway is depicted, with red crosses marking the reactions which were reduced to 0. This scheme is not a cycle anymore, but rather a chain where production of 1-InsP_7_ can only be facilitated via dephosphorylation of 1,5-InsP_8_ rather than phosphorylation of InsP_6_.

### Knockout validation of the reduced yeast model

To move beyond statistical support and test whether our reduced yeast topology is a valid biological prediction, we examined IPP concentrations in wild type, *vip1*Δ, and *kcs1*Δ strains under the same high phosphate conditions used for isotope tracing.

The reduced model predicts that the InsP${\;\;}_{6}\to$ 1-InsP_7_ branch is dispensable, implying that 1-InsP_7_ should not accumulate if Kcs1 is absent. In contrast, the canonical cyclic model predicts that Vip1 can still generate 1-InsP_7_ independently of Kcs1. Thus, the two topologies make distinct, testable predictions for the *kcs1*Δ strain.

**Fig 7:**
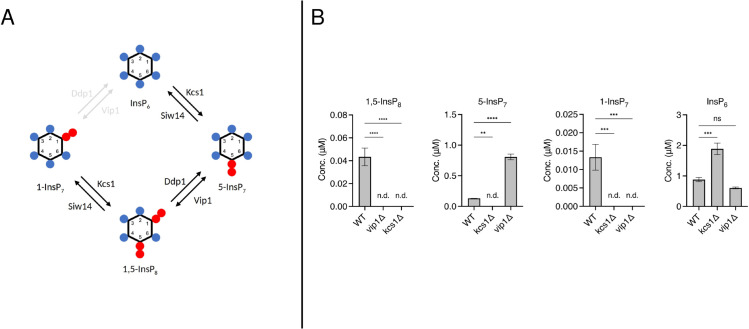
Knockout validation of the reduced yeast model. A) Schematic of the canonical cyclic pathway with enzymatic contributions of Kcs1 and Vip1. The Vip1-mediated InsP${\;\;}_{6}\to$ 1-InsP_7_ link is shown in grey, indicating that it is eliminated by model reduction and unsupported by knockout data. B) Concentrations of IPP species in wild type, *vip1*Δ, and *kcs1*Δ strains under high phosphate conditions. Values are given in *μ*M (y-axis) for each organism type (x-axis: WT, *vip1*Δ, *kcs1*Δ). Bars show mean values with error bars (± SD, *n* = 3 biological replicates). Statistical analysis was performed using Tukey’s multiple comparison test; ^****^, p < 0.0001; ^***^, p < 0.001; ^**^, p < 0.01; ^*^, p < 0.05, ns ; not significant. n.d. not detected.

As summarized in Fig 7, Kcs1 catalyzes the InsP${\;\;}_{6}\to$ 5-InsP_7_ and 1-InsP${\;\;}_{7}\to$ 1,5-InsP_8_ reactions, whereas Vip1 catalyzes InsP${\;\;}_{6}\to$ 1-InsP_7_ and 5-InsP${\;\;}_{7}\to$ 1,5-InsP_8_. Consistent with these assignments, the *vip1*Δ strain displayed the expected elevation of 5-InsP_7_ and reduction of 1,5-InsP_8_. However, the *kcs1*Δ strain exhibited no detectable 1-InsP_7_.

This outcome is difficult to reconcile with the cyclic model, which would predict 1-InsP_7_ production via Vip1, but it is fully consistent with the reduced model in which the InsP${\;\;}_{6}\to$ 1-InsP_7_ route is removed. The knockout data therefore provide independent biological evidence for the same break in the cycle predicted by the model reduction, validating the conclusion that under the tested conditions yeast IPP metabolism operates as a linear rather than cyclic pathway.

### HCT116

**Fig 8:**
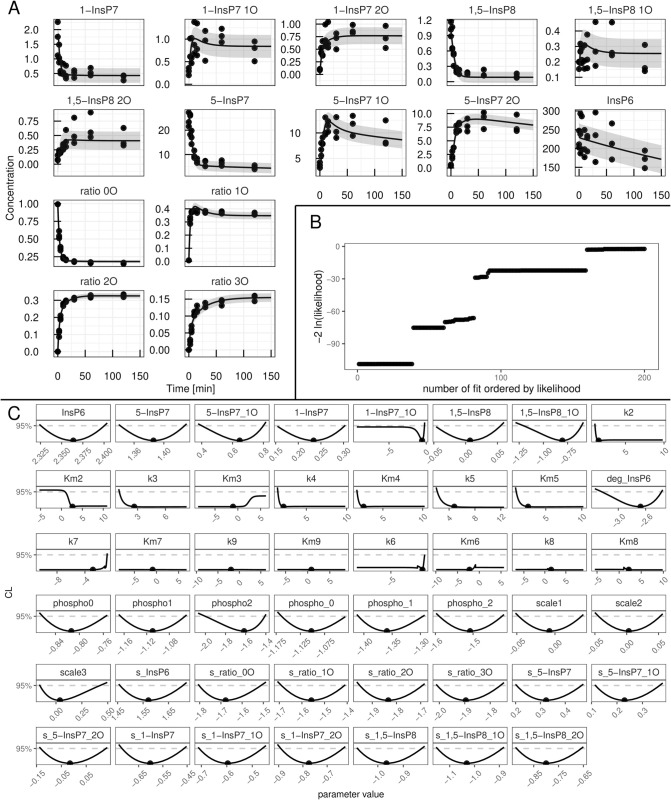
Analysis of the HCT116 data-set with the “full” mathematical model model. (A) Fit of the mathematical model with the best-fit parameters (line) to the experimental data (points). The values are given in *μ*M except for the four species in the lower left of the panel which represent ratios of 1,2,3-labelled to 0-unlabelled *γ*-phosphate. (B) Likelihood values of the 200 best multi-start fits, ordered by lowest likelihood value. (C) Profiles of the best-fit parameters of the full model (line). Best-fit parameter value are represented as points. Parameter values (x-axis) are displayed on log10 scale.

**Fig 9:**
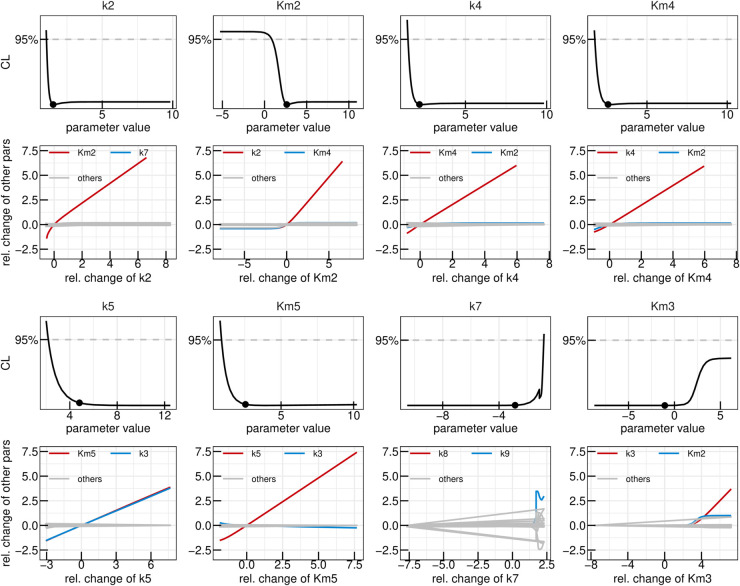
First reduction step in the HCT116 model. Profiles of the parameters considered for the first model reduction step in the yeast metabolic cycle. Best-fit parameter value are represented as points. Parameter values (x-axis) are displayed on log10 scale. Below each profile, the dependencies of all other parameters on the profiled parameter are plotted. Coupling strength is quantified by the relative change of a secondary parameter from its best-fit value after re-optimizing all parameters at each fixed value of the profiled parameter (x-axis). Both axes are shown on a log_10_ scale. The strongest dependency is highlighted in red, the next strongest in blue, and the remaining dependencies in gray.

Human HCT116 cells were grown in phosphate-replete phosphate medium and then transferred to the same phosphate medium with 50% ^18^O water. Further details on the generation of the data sets are given in Materials and Methods. The first fit with the “full” model yielded again an adequate description of the dynamics present in the data with a BIC of 164,6. The HCT116 data set together with the “full” model fit is depicted in Fig 8A. A waterfall plot displaying the sorted likelihood values of the 200 best fits is shown in Fig 8B, where eleven different initial configurations ended up in the same global optimum. Analysis of the model parameters profiles, displayed in Fig 8C, shows that also in this case a number of parameters are unidentifiable, indicated by a non-parabolic profile. Thus model reduction is required. The parameters of interest in the first model reduction step together with their respective paths can be found in Fig 9. The reactions involving *k*_2_, *k*_4_ and *k*_5_ are reduced to mass action following the reasoning established in section Yeast. Furthermore the parameters *k*_7_ and $K_{M_{3}}$ are set to zero, eliminating the transition involving *k*_7_ from the model, while reducing the *k*_3_ reaction to a zero order reaction. In a zero order reaction, as explained in section Mathematical modelling, the reaction rate is independent of the substrate concentration.

**Fig 10:**
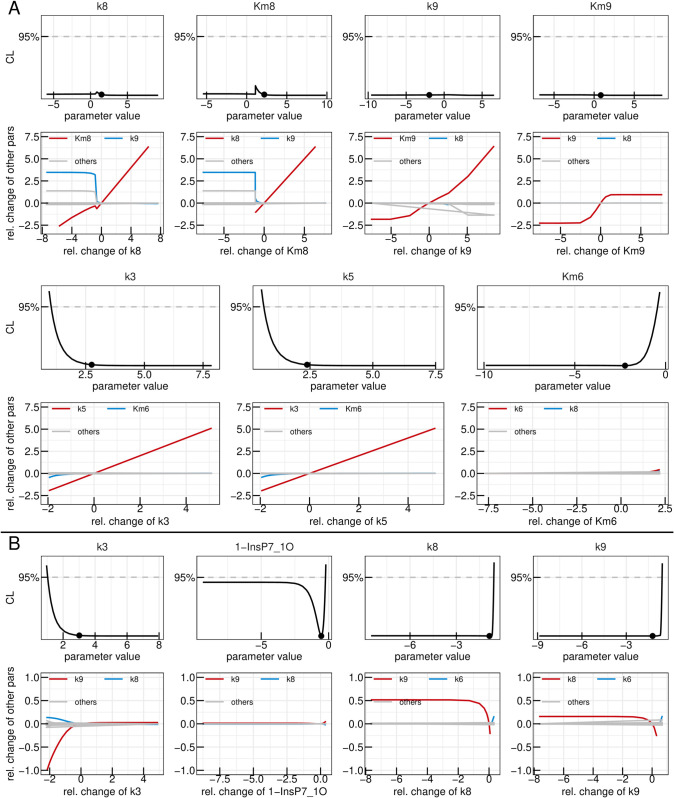
Second and third reduction steps in the HCT116 model. Profiles of the parameters considered for the second (A) and third (B) model reduction step in the yeast metabolic cycle. Best-fit parameter value are represented as points. Parameter values (x-axis) are displayed on log10 scale. Below each profile, the dependencies of all other parameters on the profiled parameter are plotted. Coupling strength is quantified by the relative change of a secondary parameter from its best-fit value after re-optimizing all parameters at each fixed value of the profiled parameter (x-axis). Both axes are shown on a log_10_ scale. The strongest dependency is highlighted in red, the next strongest in blue, and the remaining dependencies in gray.

Refitting the HCT116 data-set with the reduced model yielded no visible changes in data description but resulted in an improved BIC of 128,9. In Fig 10A, the reduction to mass-action reactions can be appreciated for the *k*_8_ and *k*_9_ transitions. Even though, $K_{M_{8}}$ shows a strong dependency on *k*_9_ for small values, for large values $K_{M_{8}}$ only depends linearly on its corresponding reaction rate, legitimising this reduction. Similar to *k*_6_ and *k*_8_ in section Yeast, here *k*_3_ and *k*_5_ depend linearly on each other while having profiles which are open towards +∞, demanding the parameter transformation $k_{5}=\frac{k_{5}}{k_{3}}\;\cdot \;k_{3}={\text{ratio}}_{k_{5},k_{3}}\cdot k_{3}$. Lastly, the reaction involving *k*_6_ is reduced to a zero order reaction following the example of *k*_3_.

This version of the model can describe the data without a visible change in goodness-of-fit, but yielded a BIC of 111,1. In a last reduction step, the parameters shown in Fig 10B are considered. *k*_3_ is set to 10^5^, while the initial value of single labelled 1-InsP_7_ is set to 0. When choosing between *k*_8_ and *k*_9_, the latter is set to zero, since it shows negligible dependence on *k*_8_ compared to vice versa.

**Fig 11:**
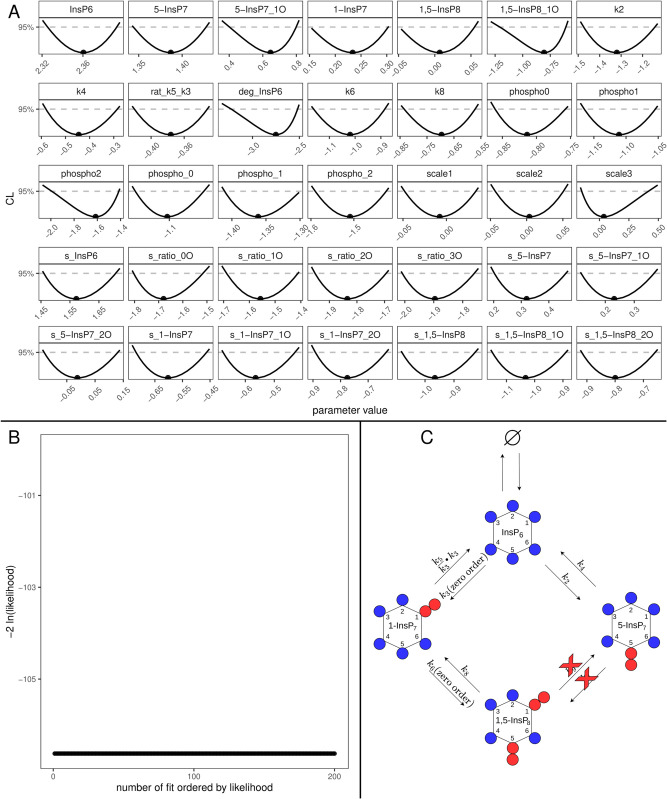
Fully reduced HCT116 model. (A) Profiles of the best-fit parameters of the reduced model (line). Best-fit parameter value are represented as points. Parameter values (x-axis) are displayed on log10 scale. (B) Likelihood values of the 200 best multi-start fits, ordered by lowest likelihood value. (C) Representation of the statistically favoured transition scheme, displaying a chain-like pattern rather than a cycle.

The final fit of the fully reduced model to the data generated a BIC of 96,94. The fully identifiable model parameters are shown in Fig 11A. In this panel, the identifiable ratio between *k*_5_ and *k*_3_ can be appreciated as well as the fact that all of the remaining reactions could be reduced to mass action while still describing the data accurately. In the final waterfall plot of the fully reduced model (Fig 11B 26 fits ended up in the global optimum. Thus also the IPP metabolic cycle of the HCT116 appears to be linear rather than cyclic (Fig 11C), where 1,5-InsP_8_ is solely generated by phosphorylation of 1-InsP_7_.

For HCT116, a second data set was generated and analysed, where the experimental conditions of the yeast experiment were copied, regarding the transition from low to high phosphate. The fits and the model reduction can be found in the supplement (S1 Fig–S4 Fig). The results are consistent with the analysis of the first HCT116 dataset in the sense that also in this reduced model, the transitions between 5-InsP_7_ and 1,5-InsP_8_ are removed. The transition between 1-InsP_7_ and InsP_6_ is also removed.

## Discussion

In this study, we investigated the metabolic pathways of inositol pyrophosphates (IPPs) in two different biological systems: yeast and the human tumour cell line HCT116. By utilising pulse-labelling experiments with ^18^O water and ordinary differential equation (ODE) models, we explored the synthesis and turnover of IPPs, aiming to clarify the pathways involved in the production of the highly phosphorylated IPP, 1,5-InsP_8_. Previous studies had proposed that 1,5-InsP_8_ could be synthesised via two distinct routes: either through 5-InsP_7_ or 1-InsP_7_ [16,29,42,55,56]. Our findings challenge this notion, suggesting that the metabolic networks in both yeast and HCT116 cells do not form a cyclic pathway but follow a linear reaction sequence.

We first applied a comprehensive (“full”) ODE model to describe the dynamics of IPP metabolism in yeast and HCT116 cells. The initial fitting results revealed that while the model could adequately capture the data, several parameters were not identifiable, indicating redundancies and dependencies that made it challenging to precisely estimate certain reaction rates and Michaelis–Menten constants. To address these issues, we performed a systematic model reduction process, heavily based on the profile-likelihood method. This process involved simplifying the model by reducing certain reactions to mass-action kinetics or even setting specific reaction rates to zero, based on their unidentifiability and lack of contribution to improving the model’s fit to the experimental data.

For the yeast system, the model reduction process led to significant insights. We found that several reactions initially assumed to be part of the metabolic cycle could be eliminated without compromising the model’s ability to describe the experimental data. Specifically, the pathway from InsP_6_ to 1,5-InsP_8_ via 1-InsP_7_ was not required, and instead, the data were best explained by a linear pathway where 1,5-InsP_8_ is produced directly from 5-InsP_7_. This finding suggests that, in yeast, the conversion of InsP_6_ to 1,5-InsP_8_ primarily occurs through the phosphorylation of 5-InsP_7_, with the dephosphorylation of 1,5-InsP_8_ being the main route for generating 1-InsP_7_.

This prediction was tested against independent experimental data. Analysis of yeast knockout strains (*vip1*Δ, *kcs1*Δ) under the same high phosphate conditions showed that the *vip1*Δ mutant accumulated 5-InsP_7_ and lost 1,5-InsP_8_, as expected, whereas the *kcs1*Δ mutant exhibited no detectable 1-InsP_7_. The latter observation is inconsistent with the canonical cyclic model, which predicts that Vip1 should provide an alternative route from InsP_6_, but it is fully consistent with the reduced linear topology. Thus, the knockout data provide biological validation of the break in the cycle predicted by model reduction.

The different reduction outcomes in yeast and human cells also provide mechanistic insight into how enzyme properties shape flux distributions *in vivo*. In yeast, model reduction consistently eliminated the InsP${\;\;}_{6}\to$ 1-InsP_7_ branch, leaving 1,5-InsP_8_ synthesis to proceed through the 5-InsP_7_ route. This agrees with *in vitro* studies showing that Vip1/PPIP5K has higher catalytic efficiency toward InsP_7_ compared to InsP_6_ [42,45,57,58], suggesting that the same preference governs flux allocation *in vivo*. By contrast, in HCT116 cells the 5-InsP${\;\;}_{7}\to$ 1,5-InsP_8_ step was eliminated, leaving 1-InsP_7_ as the principal precursor of 1,5-InsP_8_. This outcome was unexpected given existing biochemical data [42,45,55,58], and it points to species-specific differences in the relative contributions of IP6Ks and PPIP5Ks. Such differences may reflect divergent regulation of these enzymes or distinct physiological roles of 1- versus 5-InsP_7_ across eukaryotes.

In the HCT116 cell line, the analysis was more complex due to the presence of two different experimental conditions under which datasets were generated. Both conditions provided valuable insights and led to comparable yet slightly different results. The model reduction process revealed a preference for a linear rather than a cyclic metabolic pathway under both conditions. However, there were subtle differences in the parameter estimates and the specific reaction rates. While in the “normal to normal” condition, i.e. under constant ${\text{P}}_{\text{i}}$ concentration, only the transitions between 5-InsP_7_ and 1,5-InsP_8_ are removed via the model reduction approach, in the “low to high” phosphate condition, the transition from 1-InsP_7_ to InsP_6_ is additionally removed. Furthermore in the “normal to normal” condition, the conversion 1-InsP_7_ to 1,5-InsP_8_ is reduced to a zero-order reaction, while in the “high to low” setting it is the other way around. This suggests that the linear pathway might be modulated differently depending on the nutrient changes that the cells experience. Despite these differences, in both regimes the cycle is broken at the same point, reinforcing the biological relevance of this topology.

The reliance on Michaelis–Menten kinetics is another point that requires careful interpretation. This rate law cannot capture more complex behaviours such as cooperative binding or allosteric regulation. At the same time, it is a pragmatic and widely used approximation in metabolic modelling, particularly when detailed *in vivo* rate laws are unavailable. In our framework, Michaelis–Menten kinetics functions as a flexible starting point: through model reduction, the data themselves determine whether a reaction effectively reduces to mass-action, zero-order, or remains Michaelis–Menten. This allows the analysis to remain tailored to the information content of the data.

A further limitation is that statistical model reduction may discard low-flux reactions that are biologically important but experimentally difficult to resolve. However, the combination of statistical evidence and knockout validation mitigates this concern: the break predicted by the model in yeast was independently observed in the *kcs1*Δ mutant, which exhibited IPP concentrations incompatible with the cyclic model. Thus, while low-level fluxes below detection thresholds cannot be excluded, the convergence of data-driven reduction and genetic perturbation strengthens confidence in the linear topology as the major route under the studied conditions.

Finally, we took steps to ensure that the reduced models are statistically well defined and not artifacts of local minima. Before reduction, only a minority of runs reached the global optimum, while after reduction the vast majority converged to a single lowest-likelihood solution. Waterfall plots and parameter distribution plots clearly demonstrate this stabilization. In addition, the reduced models exhibit fully identifiable parameter profiles, showing that each parameter is supported by the data. These diagnostics provide strong evidence that the reduced models are robust within our modelling framework.

Interestingly, despite quantitative differences between conditions in HCT116 cells and between yeast and human cells, the consistency in the overall pathway structure underscores the robustness of the linear metabolic model. This finding challenges the traditional view of a cyclic IPP metabolic pathway and suggests that the linear pathway might be a more general feature of IPP metabolism in eukaryotic cells.

Our analysis also highlighted the importance of using both experimental data and mathematical modelling to understand complex biochemical networks. The use of pulse-labelling with ^18^O water provided dynamic information on the turnover rates of different IPPs, which was critical for parameter estimation in our ODE models. Additionally, the model reduction approach allowed us to identify the essential reactions in the metabolic pathway, leading to a more parsimonious and interpretable model that still accurately described the experimental data. Notably, in yeast the predictions from the model reduction were directly validated by independent knockout experiments, providing orthogonal support for the inferred topology.

In conclusion, this study provides new insights into the metabolic pathways of IPP conversion, revealing that the synthesis of 1,5-InsP_8_ in both yeast and HCT116 cells follows a linear rather than a cyclic pathway. The observation that different experimental conditions in HCT116 cells led to comparable, yet slightly distinct, results suggests that while the linear pathway is robust, its regulation may vary under different physiological contexts. These findings have important implications for our understanding of IPP metabolism and its regulation in eukaryotic cells. Further studies will be needed to explore the functional consequences of this linear pathway and to determine whether similar metabolic networks exist in other organisms or under varying physiological conditions.

## Materials and methods

### Mathematical modelling

The modelling approach presented in this work is based on ODEs where every dephosphorylation, or phosphorylation reaction between the considered InsP species, for all of which experimental data has been provided, was modelled according to Michaelis-Menten kinetics

$$\dot{P}=k\cdot \frac{S}{K_{m}+S}$$ (1)

where *P* is the product concentration, *S* is the substrate concentration and *K*_*M*_ is the Michaelis-Menten constant. These can be divided into three regimes, namely: **Zero-order reactions**, where the value of *K*_*M*_ is small compared to *S* and can be neglected, *S* cancels out and the reaction velocity $\dot{S}$ is independent from the substrate concentration *S*; **Michaelis-Menten reactions**, where *S* is in the range of *K*_*M*_ and the unreduced Michaelis-Menten kinetic has to be applied; **Mass-action reactions**, where *K*_*M*_ is large compared to *S* and the contribution of *S* can thus be neglected in the denominator, meaning the reaction velocity depends linearly on $\frac{k}{K_{m}}$ and *S*. *In vitro* the considered dephosphorylation, or phosphorylation reactions can be performed by multiple enzymes, but since in this work only the kinetics of the conversions as a whole are of interest, we consider the sum of the possible enzyme contributions and only model *net* reactions by a single Michaelis-Menten reaction. To account for the availability of labelled ATP and to describe the labelling of the different molecules, the phosphorylation reaction velocities $k_{\text{i}}$ are multiplied with the relative abundance of the labelled ATP $ratio_{\text{XO}}$ where X∈{0,1,2,3} designates the number of labelled oxygen atoms in the *γ*-phosphate, for which experimental data is also available. In this analysis we considered two cell types, namely baker’s yeast and human HCT116 cells which were both analysed with the identical initial model consisting of 29 states and 48 dynamic parameters. The reactions of the initial full model are shown in Table 1.

**Table 1:** Reactions of the full unreduced mathematical model. The reactions are based on Michaelis-Menten and mass-action kinetics, where X and X’ specify the number of heavy labelled oxygen in the respective phosphate groups, with X and X’∈{0,1,2,3}.

Educt	Product	Rate
∅	${\text{InsP}}_{6}$	${\text{prod}}_{{\text{InsP}}_{6}}$
${\text{InsP}}_{6}$	∅	${\text{deg}}_{{\text{InsP}}_{6}}\cdot {\text{InsP}}_{6}$
${\text{InsP}}_{6}$	${\text{5-InsP}}_{7,\text{XO}}$	$\frac{k_{2}\cdot {\text{ratio}}_{\text{XO}}\cdot {\text{InsP}}_{6}}{Km_{2}+{\text{InsP}}_{6}}$
${\text{InsP}}_{6}$	${\text{1-InsP}}_{7,\text{XO}}$	$\frac{k_{3}\cdot {\text{ratio}}_{\text{XO}}\cdot {\text{InsP}}_{6}}{Km_{3}+{\text{InsP}}_{6}}$
${\text{5-InsP}}_{7,\text{XO}}$	${\text{InsP}}_{6}$	$\frac{k_{4}\cdot {\text{5-InsP}}_{7,\text{XO}}}{Km_{4}+{\text{5-InsP}}_{7,\text{XO}}}$
${\text{1-InsP}}_{7,\text{XO}}$	${\text{InsP}}_{6}$	$\frac{k_{5}\cdot {\text{1-InsP}}_{7,\text{XO}}}{Km_{5}+{\text{1-InsP}}_{7,\text{XO}}}$
${\text{1-InsP}}_{7,\text{XO}}$	${\text{1,5-InsP}}_{8,\text{XOX'O}}$	$\frac{k_{6}\cdot {\text{1-InsP}}_{7,\text{XO}}\cdot {\text{ratio}}_{\text{X'O}}}{Km_{6}+{\text{1-InsP}}_{7,\text{XO}}}$
${\text{5-InsP}}_{7,\text{XO}}$	${\text{1,5-InsP}}_{\text{8,1X'O+5XO}}$	$\frac{k_{7}\cdot {\text{5-InsP}}_{7,\text{XO}}\cdot {\text{ratio}}_{\text{X'O}}}{Km_{7}+{\text{5-InsP}}_{7,\text{XO}}}$
${\text{1,5-InsP}}_{8,\text{XOX'O}}$	${\text{1-InsP}}_{7,\text{XO}}$	$\frac{k_{8}\cdot {\text{1,5-InsP}}_{8,\text{XOX'O}}}{Km_{8}+{\text{1,5-InsP}}_{8,\text{XOX'O}}}$
${\text{1,5-InsP}}_{8,\text{XOX'O}}$	${\text{5-InsP}}_{7,\text{X'O}}$	$\frac{k_{9}\cdot {\text{1,5-InsP}}_{8,\text{XOX'O}}}{Km_{9}+{\text{1,5-InsP}}_{8,\text{XOX'O}}}$
${\text{ratio}}_{\text{0O}}$	${\text{ratio}}_{\text{1O}}$	${\text{phospho}}_{0}\cdot {\text{ratio}}_{\text{0O}}$
${\text{ratio}}_{\text{0O}}$	${\text{ratio}}_{\text{2O}}$	${\text{phospho}}_{1}\cdot {\text{ratio}}_{\text{0O}}$
${\text{ratio}}_{\text{0O}}$	${\text{ratio}}_{\text{3O}}$	${\text{phospho}}_{2}\cdot {\text{ratio}}_{\text{0O}}$
${\text{ratio}}_{\text{1O}}$	${\text{ratio}}_{\text{0O}}$	${\text{phospho}}_{-0}\cdot {\text{ratio}}_{\text{1O}}$
${\text{ratio}}_{\text{2O}}$	${\text{ratio}}_{\text{0O}}$	${\text{phospho}}_{-1}\cdot {\text{ratio}}_{\text{2O}}$
${\text{ratio}}_{\text{3O}}$	${\text{ratio}}_{\text{0O}}$	${\text{phospho}}_{-2}\cdot {\text{ratio}}_{\text{3O}}$

An absolute error model was assumed, with error parameters estimated simultaneously with the kinetic parameters. To assess the validity of this assumption, we performed a residuals-versus-prediction analysis for the yeast dataset (S5 Fig). The plot shows no systematic fanning of residuals with increasing predicted values, supporting the use of an absolute error model. Because the same measurement technique was applied in all datasets, this validation was performed once and taken to apply across all remaining datasets. The estimated errors are depicted as grey bands around the plotted predictions for every dataset. The observables and corresponding error parameters are summarized in Table 2. For all parameters, a logarithmic transformation was performed prior to optimization to ensure positivity and to allow efficient sampling across a wide dynamic range.

**Table 2:** Observables and error parameters of the full unreduced mathematical model.

Dataset	Observable	Error parameter
${\text{InsP}}_{6}$	${\text{InsP}}_{6}$	$\text{s\_}{\text{InsP}}_{6}$
${\text{5-InsP}}_{7}$	${\text{5-InsP}}_{7}$	$\text{s\_}{\text{5-InsP}}_{7}$
${\text{5-InsP}}_{\text{7,1O}}$	${\text{5-InsP}}_{\text{7,1O}}$	$\text{s\_}{\text{5-InsP}}_{\text{7,1O}}$
${\text{5-InsP}}_{\text{7,2O}}$	${\text{5-InsP}}_{\text{7,2O}}$	$\text{s\_}{\text{5-InsP}}_{\text{7,2O}}$
${\text{1-InsP}}_{7}$	${\text{1-InsP}}_{7}$	$\text{s\_}{\text{1-InsP}}_{7}$
${\text{1-InsP}}_{\text{7,1O}}$	${\text{1-InsP}}_{\text{7,1O}}$	$\text{s\_}{\text{1-InsP}}_{\text{7,1O}}$
${\text{1,5-InsP}}_{8}$	${\text{1,5-InsP}}_{8}$	$\text{s\_}{\text{1,5-InsP}}_{8}$
${\text{1,5-InsP}}_{\text{8,1O}}$	${\text{1,5-InsP}}_{\text{8,1O0O}}+{\text{1,5-InsP}}_{\text{8,0O1O}}$	$\text{s\_}{\text{1,5-InsP}}_{\text{8,1O}}$
${\text{1,5-InsP}}_{\text{8,2O}}$	${\text{1,5-InsP}}_{\text{8,2O0O}}+{\text{1,5-InsP}}_{\text{8,0O2O}}+{\text{1,5-InsP}}_{\text{8,1O1O}}$	$\text{s\_}{\text{1,5-InsP}}_{\text{8,2O}}$
${\text{ratio}}_{\text{0O}}$	${\text{ratio}}_{\text{0O}}$	$\text{s\_}{\text{ratio}}_{\text{0O}}$
${\text{ratio}}_{\text{1O}}$	${\text{scale}}_{1}\cdot {\text{ratio}}_{\text{1O}}$	$\text{s\_}{\text{ratio}}_{\text{1O}}$
${\text{ratio}}_{\text{2O}}$	${\text{scale}}_{2}\cdot {\text{ratio}}_{\text{2O}}$	$\text{s\_}{\text{ratio}}_{\text{2O}}$
${\text{ratio}}_{\text{3O}}$	${\text{scale}}_{3}\cdot {\text{ratio}}_{\text{3O}}$	$\text{s\_}{\text{ratio}}_{\text{3O}}$

### Parameter estimation and selection of mathematical models

In order to determine the optimal parameter set, the local deterministic Gauss-Newton gradient-based trust-region optimiser with a tolerance setting of 10^−10^ was used, which is implemented in the *R* package *dMod* [47]. To ensure, that the “best” parameter set which is found is also the globally optimal set, a multi-start analyses with 1000 random initial parameters sets was performed. To determine how well a fit describes the analysed data the Bayesian information criterion (BIC) is used in this work. The BIC is defined as BIC=kln(n)−2ln(ℒ), where *k* is the number of model parameters, *n* is the number of data points and ℒ is the maximised value of the likelihood function. Lower BIC values indicate preferable description of the analysed data. Thus, when two models describe the same data comparably well, the model with the lower number of parameters, following Occam’s razor, is to be preferred. It should be mentioned that there exists another popular metric of comparing two models called Akaike information criterion (AIC), defined as AIC=2k−2ln(ℒ). However, in this work we use BIC since it is a more stringent criterion and AIC under-penalizes the number of parameters if *n* becomes large. It should be mentioned, that the BIC value is only useful as a relative comparison between different models, while the absolute value of the BIC has no real meaning for any given model.

### Profile likelihood

Analysing parameter uncertainties as well as identifiability of the latter is a key task in mathematical modelling. While there are other methods, like Markov-Chain-Monte-Carlo methods (MCMC), to analyse parameter uncertainty, in this project the profile likelihood method was employed [49,50]. The profile likelihood is a Likelihood ratio test with one degree of freedom, where the analysed parameter is slightly changed from its best-fit value and all remaining parameters are re-optimised. The ratio between the initial best likelihood value and the new one is then plotted. This process is performed multiple times in a set interval for the analysed parameter, usually +/-3 around the best-fit value on log-scale, and ideally will then give a bell shaped curve passing a 95% confidence level threshold for larger and smaller values of the best-fit value of the parameter. This method cannot only be used to investigate whether model parameters are identifiable, but also provides statistically sound estimates for parameter uncertainties. Furthermore, this method can also guide model reduction by providing not only the parameter uncertainties but also their dependencies on the other parameters in the model. The dependencies depicted under the profile likelihood plots show the relative change of the other parameters from their best-fit value as a function of the relative change of the profiled parameter’s value from its best-fit estimate after refitting. To perform the uncertainty analysis in *R*, the profile likelihood function of the *dMod* package was used.

### Yeast strains and genetic manipulation

The Saccharomyces cerevisiae BY4741 was used in this study. The plasmid used to overexpress the truncated Pho90 (Pho90^ΔSPX^) was generated by cloning the Pho90 open reading frame excluding N-terminal 375 amino acids between BamHI and XhoI restriction enzyme sites of the parent plasmid (pRS415) (Mumberg, Muller, and Funk, 1995) containing TEF1 promoter. For DNA transformation, BY4741 strain was logarithmically grown at 30 °C in 50 mL of YPD (yeast extract-peptone-dextrose) medium. Cultures (4.6 x 10^7^ cells/mL) were centrifuged at 3200 g for 3 min. The pellet was washed with the same volume of TE buffer (10 mM of Tris-HCl pH 7.5 and 1 mM of EDTA) and resuspended with 10 mL of LiAC-TE buffer (TE buffer with 100 mM of LiAC). After the incubation at room temperature for 15 min, cells were collected by centrifugation at 3,000 g for 3 min. The pellet was resuspended with 800 *μ*L of LiAC-TE buffer. 40 *μ*L of cells were mixed with 200 ng of plasmid DNA, 5 *μ*L of salmon sperm DNA (10 mg/mL) and 300 *μ*L of PEG-LiAC-TE buffer (50% of polyethylene glycol 4000). After 20 min at 30 °C with gentle shaking, a heat shock was carried out at 42 °C for 20 min with gentle shaking. Cells were centrifuged at 3,000 g for 2 min, washed twice with water, and grown on selection plates for 2-4 days at 30 °C. The *kcs1*Δ and *vip1*Δ mutants were obtained from the strains used in [29]. For yeast growth, synthetic complete (SC) medium was prepared from yeast nitrogen base without phosphate (Formedium, UK). The phosphate concentration was adjusted with KH_2_PO_4_. Potassium concentration was controlled by adding KCl instead of KH_2_PO_4_.

### Extraction of IPPs and ATP from yeast cells

Yeast strain overexpressing Pho90^ΔSPX^ was grown at 20 °C in SC medium containing 0.2 mM of phosphate until logarithmic phase (4.6 x 10^7^ cells/mL). Samples were harvested by centrifugation at 3,000 g for 3 min and resuspended in the same volume of SC medium containing 50 mM of phosphate prepared with 50 % of ^18^O-labelled water. Yeast cells were further incubated at 20 °C. At each time point, 3 ml of yeast culture was mixed with 300 *μ*l of 11 M perchloric acid to a final concentration of 1 M and snap frozen in liquid nitrogen. IPPs and ATP were extracted from yeast samples based on [52]. Thawed samples were centrifuged at 20,000 g for 3 min at 4 °C and the soluble supernatant was transferred into a new tube. 6 mg of titanium dioxide (TiO_2_) beads per sample (GL Sciences, Japan) was washed twice with 1 mL of water and 1 M of perchloric acid respectively, and then mixed with the soluble supernatant [59]. The mixture was gently rotated for 15 min at 4 °C and centrifuged at 20,000 g for 3 min at 4 °C. After two rounds of washing with 500 *μ*L of 1 M perchloric acid, the TiO_2_ beads were incubated with 300 *μ*L of 3 % (v/v) NH_4_OH at 25 °C for 5 min with gently shaking to elute IPPs and ATP. The samples were centrifuged at 20,000 g for 1 min and the eluents were transferred into a new tube. The additional centrifugation was conducted to completely remove the residual TiO_2_ beads from the eluents. The resulting supernatant was dried in a SpeedVac (Labogene, Denmark) at 42 °C. Samples were kept at -20 °C until analysis.

For mutant analysis, cells were grown in SC medium to logarithimic phase. A total of 5 OD_600_ units of cells were harvested by centrifugation at 3,000 g for 2 min, resuspended in 1 mL of 1 M perchloric acid, and snap-frozen in liquid nitrogen. IPPs were then extracted as described above. Dried samples were reconstituted in 30 *μ*L of water before analysis.

All time points were generated in three biological replicates.

### Mammalian cell culture and extraction of IPPs and ATP

IPPs and ATP data from HCT116 cells under normal-to-normal phosphate (P_i_) conditions were obtained from our previously published work [52], while data under low-to-high P_i_ conditions were generated in the present study. The cell culture preparation and IPP extraction were performed following similar procedures with modifications as described in our previous work. HCT116 cells were cultured for six passages in complete DMEM (Thermo Fisher Scientific, Cat. No. 31966021) supplemented with 10% (v/v) fetal bovine serum (PAN Biotech, Cat. No. P30-3306) and 100 U/mL penicillin-streptomycin (Thermo Fisher Scientific). Cells were grown at 37°C in a humidified incubator with 5% CO_2_ and 98% humidity. The low phosphate medium (0.05 mM P_i_) was prepared using P_i_-free DMEM (Thermo Fisher Scientific, Cat. No. 11971025) supplemented with 100 U/mL penicillin-streptomycin, 1 mM sodium pyruvate, and 0.05 mM phosphate (NaH_2_PO_4_). For high phosphate medium containing 50% ^18^O water, a 2x concentrated medium was prepared from DMEM powder (Sigma, Cat. No. D5648) supplemented with 100 U/mL penicillin-streptomycin, 28.16 mM P_i_ (total 30 mM P_i_, including 1.84 mM P_i_ from the DMEM), and 7.4 g/L sodium bicarbonate. This 2x medium was then diluted with an equal volume of ^18^O-labelled water to yield a final medium containing 50% ^18^O water. For the low-to-high P_i_ experiments, HCT116 cells were seeded in complete DMEM and allowed to adhere for 24 hours. Upon reaching approximately 80% confluency, the cells were cultured in low P_i_ medium (0.05 mM P_i_) for 24 hours, and then shifted to high P_i_ medium (15 mM P_i_) prepared with 50% ^18^O water. At each time point, the medium was removed, and the cells were harvested in 5 mL of 1 M perchloric acid. After snap-freezing in liquid nitrogen, the samples were centrifuged at 3,200 xg for 5 minutes. The soluble supernatant was transferred to a new tube and mixed with TiO_2_ beads (5 mg per sample). IPPs and ATP were extracted as described previously for yeast cells. All time points were generated in three biological replicates.

## Supporting information

S1 Fig Analysis of the HCT116 data-set with the “full” mathematical model model. (A) Fit of the mathematical model with the best-fit parameters (line) to the experimental data (points). The values are given in *μ*M except for the four species in the lower left of the panel which represent ratios of 1,2,3-labelled to 0-unlabelled *γ*-phosphate. (B) Likelihood values of the 200 best multi-start fits, ordered by lowest likelihood value. (C) Profiles of the best-fit parameters of the full model (line). Best-fit parameter value are represented as points. Parameter values (x-axis) are displayed on log10 scale.
(TIFF)

S2 Fig First reduction step in the HCT116 model. Profiles of the parameters considered for the first model reduction step in the yeast metabolic cycle. Best-fit parameter value are represented as points. Parameter values (x-axis) are displayed on log10 scale. Below each profile, the dependencies of all other parameters on the profiled parameter are plotted. Coupling strength is quantified by the relative change of a secondary parameter from its best-fit value after re-optimizing all parameters at each fixed value of the profiled parameter (x-axis). Both axes are shown on a log_10_ scale. The strongest dependency is highlighted in red, the next strongest in blue, and the remaining dependencies in gray.
(TIFF)

S3 Fig Second and third reduction steps in the HCT116 model. Profiles of the parameters considered for the second (A) and third (B) model reduction step in the yeast metabolic cycle. Best-fit parameter value are represented as points. Parameter values (x-axis) are displayed on log10 scale.Below each profile, the dependencies of all other parameters on the profiled parameter are plotted. Coupling strength is quantified by the relative change of a secondary parameter from its best-fit value after re-optimizing all parameters at each fixed value of the profiled parameter (x-axis). Both axes are shown on a log_10_ scale. The strongest dependency is highlighted in red, the next strongest in blue, and the remaining dependencies in gray.
(TIFF)

S4 Fig Fully reduced HCT116 model. (A) Profiles of the best-fit parameters of the reduced model (line). Best-fit parameter value are represented as points. Parameter values (x-axis) are displayed on log10 scale. (B) Likelihood values of the 200 best multi-start fits, ordered by lowest likelihood value. (C) Representation of the statistically favoured transition scheme, displaying a chain-like pattern rather than a cycle, with an additional removed transition between 1-InsP_7_ and InsP_6_ compared to the normal-to-normal HCT116 data set.
(TIFF)

S5 Fig Absolute residuals (value of datapoint minus value of its prediction) plotted against predicted values for each observable. Dashed horizontal lines indicate zero residuals. A roughly constant spread of residuals across the range of predictions supports the assumption of homoscedasticity, and thus the use of an absolute error model.
(TIFF)

S6 Fig Absolute residuals (value of datapoint minus value of its prediction) plotted against time-points for each observable. Dashed horizontal lines indicate zero residuals. A roughly constant spread of residuals across the range of time-points shows that fit is not unduly driven by certain noisy time-points.
(TIFF)

S7 Fig Parameter distributions of the 200 best fits in the lowest step of the reduced yeast model. Parameter values are displayed on log10 scale and follow a point-like distribution with no discernable variance over 200 fits.
(TIFF)

S8 Fig Parameter distributions of the 200 best fits in the lowest step of the reduced HCT116 normal to normal P_i_ model. Parameter values are displayed on log10 scale and follow a point-like distribution with no discernable variance over 200 fits.
(TIFF)

S9 Fig Parameter distributions of the 200 best fits in the lowest step of the reduced HCT116 low to high P_i_ model. Parameter values are displayed on log10 scale and follow a point-like distribution with no discernable variance over 200 fits.
(TIFF)

## References

[1] Holub BJ. Metabolism and function of myo-inositol and inositol phospholipids. Annu Rev Nutr. 1986;6:563–97. doi: 10.1146/annurev.nu.06.070186.003023 2425833

[2] Wild R, Gerasimaite R, Jung J-Y, Truffault V, Pavlovic I, Schmidt A, et al. Control of eukaryotic phosphate homeostasis by inositol polyphosphate sensor domains. Science. 2016;352(6288):986–90. doi: 10.1126/science.aad9858 27080106

[3] Ried MK, Wild R, Zhu J, Pipercevic J, Sturm K, Broger L, et al. Inositol pyrophosphates promote the interaction of SPX domains with the coiled-coil motif of PHR transcription factors to regulate plant phosphate homeostasis. Nat Commun. 2021;12(1):384. doi: 10.1038/s41467-020-20681-4 33452263 PMC7810988

[4] Pipercevic J, Kohl B, Gerasimaite R, Comte-Miserez V, Hostachy S, Müntener T, et al. Inositol pyrophosphates activate the vacuolar transport chaperone complex in yeast by disrupting a homotypic SPX domain interaction. Nat Commun. 2023;14(1):2645. doi: 10.1038/s41467-023-38315-w 37156835 PMC10167327

[5] Liu W, Wang J, Comte-Miserez V, Zhang M, Yu X, Chen Q, et al. Cryo-EM structure of the polyphosphate polymerase VTC reveals coupling of polymer synthesis to membrane transit. EMBO J. 2023;42(10):e113320. doi: 10.15252/embj.2022113320 37066886 PMC10183816

[6] Lu Y, Yue C-X, Zhang L, Yao D, Xia Y, Zhang Q, et al. Structural basis for inositol pyrophosphate gating of the phosphate channel XPR1. Science. 2024;386(6723):eadp3252. doi: 10.1126/science.adp3252 39325866

[7] Yan R, Chen H, Liu C, Zhao J, Wu D, Jiang J, et al. Human XPR1 structures reveal phosphate export mechanism. Nature. 2024;633(8031):960–7. doi: 10.1038/s41586-024-07852-9 39169184

[8] Chakraborty A. The inositol pyrophosphate pathway in health and diseases. Biol Rev Camb Philos Soc. 2018;93(2):1203–27. doi: 10.1111/brv.12392 29282838 PMC6383672

[9] Thota SG, Bhandari R. The emerging roles of inositol pyrophosphates in eukaryotic cell physiology. J Biosci. 2015;40(3):593–605. doi: 10.1007/s12038-015-9549-x 26333405

[10] Austin S, Mayer A. Phosphate homeostasis - a vital metabolic equilibrium maintained through the INPHORS signaling pathway. Front Microbiol. 2020;11:1367. doi: 10.3389/fmicb.2020.01367 32765429 PMC7381174

[11] Shears SB. Intimate connections: Inositol pyrophosphates at the interface of metabolic regulation and cell signaling. J Cell Physiol. 2018;233(3):1897–912. doi: 10.1002/jcp.26017 28542902 PMC5694711

[12] Lee S, Kim MG, Ahn H, Kim S. Inositol pyrophosphates: signaling molecules with pleiotropic actions in mammals. Molecules. 2020;25. 10.3390/molecules25092208 PMC7249018 32397291

[13] Qi J, Shi L, Zhu L, Chen Y, Zhu H, Cheng W. Functions, mechanisms, and therapeutic applications of the inositol pyrophosphates 5PP-InsP(5) and InsP(8) in mammalian cells. J Cardiovasc Transl Res. 2023. 10.1007/s12265-023-10427-0 37615888

[14] Saiardi A, Erdjument-Bromage H, Snowman AM, Tempst P, Snyder SH. Synthesis of diphosphoinositol pentakisphosphate by a newly identified family of higher inositol polyphosphate kinases. Curr Biol. 1999;9(22):1323–6. doi: 10.1016/s0960-9822(00)80055-x 10574768

[15] Saiardi A, Nagata E, Luo HR, Snowman AM, Snyder SH. Identification and characterization of a novel inositol hexakisphosphate kinase. J Biol Chem. 2001;276(42):39179–85. doi: 10.1074/jbc.M106842200 11502751

[16] Mulugu S, Bai W, Fridy PC, Bastidas RJ, Otto JC, Dollins DE, et al. A conserved family of enzymes that phosphorylate inositol hexakisphosphate. Science. 2007;316(5821):106–9. doi: 10.1126/science.1139099 17412958

[17] Fridy PC, Otto JC, Dollins DE, York JD. Cloning and characterization of two human VIP1-like inositol hexakisphosphate and diphosphoinositol pentakisphosphate kinases. J Biol Chem. 2007;282(42):30754–62. doi: 10.1074/jbc.M704656200 17690096

[18] Gaugler P, Schneider R, Liu G, Qiu D, Weber J, Schmid J, et al. Arabidopsis PFA-DSP-type phosphohydrolases target specific inositol pyrophosphate messengers. Biochemistry. 2022;61(12):1213–27. doi: 10.1021/acs.biochem.2c00145 35640071 PMC9351621

[19] Qiu D, Gu C, Liu G, Ritter K, Eisenbeis VB, Bittner T, et al. Capillary electrophoresis mass spectrometry identifies new isomers of inositol pyrophosphates in mammalian tissues. Chem Sci. 2022;14(3):658–67. doi: 10.1039/d2sc05147h 36741535 PMC9847636

[20] Dollins DE, Bai W, Fridy PC, Otto JC, Neubauer JL, Gattis SG, et al. Vip1 is a kinase and pyrophosphatase switch that regulates inositol diphosphate signaling. Proc Natl Acad Sci U S A. 2020;117(17):9356–64. doi: 10.1073/pnas.1908875117 32303658 PMC7196807

[21] Wang H, Nair VS, Holland AA, Capolicchio S, Jessen HJ, Johnson MK, et al. Asp1 from Schizosaccharomyces pombe binds a [2Fe-2S](2+) cluster which inhibits inositol pyrophosphate 1-phosphatase activity. Biochemistry. 2015;54(42):6462–74. doi: 10.1021/acs.biochem.5b00532 26422458 PMC4641441

[22] Safrany ST, Caffrey JJ, Yang X, Bembenek ME, Moyer MB, Burkhart WA, et al. A novel context for the “MutT” module, a guardian of cell integrity, in a diphosphoinositol polyphosphate phosphohydrolase. EMBO J. 1998;17(22):6599–607. doi: 10.1093/emboj/17.22.6599 9822604 PMC1171006

[23] Safrany ST, Ingram SW, Cartwright JL, Falck JR, McLennan AG, Barnes LD, et al. The diadenosine hexaphosphate hydrolases from Schizosaccharomyces pombe and Saccharomyces cerevisiae are homologues of the human diphosphoinositol polyphosphate phosphohydrolase. Overlapping substrate specificities in a MutT-type protein. J Biol Chem. 1999;274(31):21735–40. doi: 10.1074/jbc.274.31.21735 10419486

[24] Kilari RS, Weaver JD, Shears SB, Safrany ST. Understanding inositol pyrophosphate metabolism and function: kinetic characterization of the DIPPs. FEBS Lett. 2013;587(21):3464–70. doi: 10.1016/j.febslet.2013.08.035 24021644 PMC3873756

[25] Márquez-Moñino MÁ, Ortega-García R, Shipton ML, Franco-Echevarría E, Riley AM, Sanz-Aparicio J, et al. Multiple substrate recognition by yeast diadenosine and diphosphoinositol polyphosphate phosphohydrolase through phosphate clamping. Sci Adv. 2021;7(17):eabf6744. doi: 10.1126/sciadv.abf6744 33893105 PMC8064635

[26] Steidle EA, Chong LS, Wu M, Crooke E, Fiedler D, Resnick AC, et al. A novel inositol pyrophosphate phosphatase in Saccharomyces cerevisiae: Siw14 protein selectively cleaves the *β*-phosphate from 5-diphosphoinositol pentakisphosphate (5PP-IP5). J Biol Chem. 2016;291(13):6772–83. doi: 10.1074/jbc.M116.714907 26828065 PMC4807264

[27] Benjamin B, Garg A, Jork N, Jessen HJ, Schwer B, Shuman S. Activities and structure-function analysis of fission yeast Inositol Pyrophosphate (IPP) kinase-pyrophosphatase Asp1 and its impact on regulation of pho1 gene expression. mBio. 2022;13(3):e0103422. doi: 10.1128/mbio.01034-22 35536002 PMC9239264

[28] Qin N, Li L, Ji X, Pereira R, Chen Y, Yin S, et al. Flux regulation through glycolysis and respiration is balanced by inositol pyrophosphates in yeast. Cell. 2023;186(4):748-763.e15. doi: 10.1016/j.cell.2023.01.014 36758548

[29] Chabert V, Kim G-D, Qiu D, Liu G, Michaillat Mayer L, Jamsheer K M, et al. Inositol pyrophosphate dynamics reveals control of the yeast phosphate starvation program through 1,5-IP8 and the SPX domain of Pho81. Elife. 2023;12:RP87956. doi: 10.7554/eLife.87956 37728314 PMC10511240

[30] Menniti FS, Miller RN, Putney JW Jr, Shears SB. Turnover of inositol polyphosphate pyrophosphates in pancreatoma cells.. Journal of Biological Chemistry. 1993;268(6):3850–6. doi: 10.1016/s0021-9258(18)53551-1 8382679

[31] Dong J, Ma G, Sui L, Wei M, Satheesh V, Zhang R, et al. Inositol pyrophosphate InsP8 acts as an intracellular phosphate signal in arabidopsis. Mol Plant. 2019;12(11):1463–73. doi: 10.1016/j.molp.2019.08.002 31419530

[32] Li X, Gu C, Hostachy S, Sahu S, Wittwer C, Jessen HJ, et al. Control of XPR1-dependent cellular phosphate efflux by InsP8 is an exemplar for functionally-exclusive inositol pyrophosphate signaling. Proc Natl Acad Sci U S A. 2020;117(7):3568–74. doi: 10.1073/pnas.1908830117 32019887 PMC7035621

[33] Barker CJ, Wright J, Hughes PJ, Kirk CJ, Michell RH. Complex changes in cellular inositol phosphate complement accompany transit through the cell cycle. Biochem J. 2004;380(Pt 2):465–73. doi: 10.1042/BJ20031872 14992690 PMC1224188

[34] Banfic H, Bedalov A, York JD, Visnjic D. Inositol pyrophosphates modulate S phase progression after pheromone-induced arrest in Saccharomyces cerevisiae. J Biol Chem. 2013;288(3):1717–25. doi: 10.1074/jbc.M112.412288 23179856 PMC3548482

[35] Bru S, Martínez-Laínez JM, Hernández-Ortega S, Quandt E, Torres-Torronteras J, Martí R, et al. Polyphosphate is involved in cell cycle progression and genomic stability in Saccharomyces cerevisiae. Mol Microbiol. 2016;101(3):367–80. doi: 10.1111/mmi.13396 27072996

[36] Neef DW, Kladde MP. Polyphosphate loss promotes SNF/SWI- and Gcn5-dependent mitotic induction of PHO5. Mol Cell Biol. 2003;23(11):3788–97. doi: 10.1128/MCB.23.11.3788-3797.2003 12748282 PMC155216

[37] Kuenzel NA, Alcazar-Roman AR, Saiardi A, Bartsch SM, Daunaraviciute S, Fiedler D. Inositol pyrophosphate-controlled kinetochore architecture and mitotic entry in S. pombe. J Fungi (Basel). 2022;8. 10.3390/jof8090933 PMC9506091 36135658

[38] Pesesse X, Choi K, Zhang T, Shears SB. Signaling by higher inositol polyphosphates. Synthesis of bisdiphosphoinositol tetrakisphosphate (“InsP8”) is selectively activated by hyperosmotic stress. J Biol Chem. 2004;279(42):43378–81. doi: 10.1074/jbc.C400286200 15316027

[39] Steidle EA, Morrissette VA, Fujimaki K, Chong L, Resnick AC, Capaldi AP, et al. The InsP7 phosphatase Siw14 regulates inositol pyrophosphate levels to control localization of the general stress response transcription factor Msn2. J Biol Chem. 2020;295(7):2043–56. doi: 10.1074/jbc.RA119.012148 31848224 PMC7029108

[40] Eisenbeis VB, Qiu D, Gorka O, Strotmann L, Liu G, Prucker I, et al. *β*-lapachone regulates mammalian inositol pyrophosphate levels in an NQO1- and oxygen-dependent manner. Proc Natl Acad Sci U S A. 2023;120(34):e2306868120. doi: 10.1073/pnas.2306868120 37579180 PMC10450438

[41] Choi K, Mollapour E, Shears SB. Signal transduction during environmental stress: InsP8 operates within highly restricted contexts. Cell Signal. 2005;17(12):1533–41. doi: 10.1016/j.cellsig.2005.03.021 15936174

[42] Randall TA, Gu C, Li X, Wang H, Shears SB. A two-way switch for inositol pyrophosphate signaling: Evolutionary history and biological significance of a unique, bifunctional kinase/phosphatase. Adv Biol Regul. 2020;75:100674. doi: 10.1016/j.jbior.2019.100674 31776069 PMC9383039

[43] Glennon MC, Shears SB. Turnover of inositol pentakisphosphates, inositol hexakisphosphate and diphosphoinositol polyphosphates in primary cultured hepatocytes. Biochem J. 1993;293 (Pt 2)(Pt 2):583–90. doi: 10.1042/bj2930583 8343137 PMC1134401

[44] Weaver JD, Wang H, Shears SB. The kinetic properties of a human PPIP5K reveal that its kinase activities are protected against the consequences of a deteriorating cellular bioenergetic environment. Biosci Rep. 2013;33(2):e00022. doi: 10.1042/BSR20120115 23240582 PMC3564036

[45] Gu C, Nguyen H-N, Hofer A, Jessen HJ, Dai X, Wang H, et al. The significance of the bifunctional kinase/phosphatase activities of diphosphoinositol pentakisphosphate kinases (PPIP5Ks) for coupling inositol pyrophosphate cell signaling to cellular phosphate homeostasis. J Biol Chem. 2017;292(11):4544–55. doi: 10.1074/jbc.M116.765743 28126903 PMC5377771

[46] Gu C, Wilson MSC, Jessen HJ, Saiardi A, Shears SB. Inositol pyrophosphate profiling of two HCT116 cell lines uncovers variation in InsP8 levels. PLoS One. 2016;11(10):e0165286. doi: 10.1371/journal.pone.0165286 27788189 PMC5082907

[47] Kaschek D, Mader W, Fehling-Kaschek M, Rosenblatt M, Timmer J. Dynamic modeling, parameter estimation, and uncertainty analysis in R. J Stat Soft. 2019;88(10). doi: 10.18637/jss.v088.i10

[48] Raue A, Schilling M, Bachmann J, Matteson A, Schelker M, Kaschek D, et al. Lessons learned from quantitative dynamical modeling in systems biology. PLoS One. 2013;8(9):e74335. doi: 10.1371/journal.pone.0074335 24098642 PMC3787051

[49] Kreutz C, Raue A, Kaschek D, Timmer J. Profile likelihood in systems biology. FEBS J. 2013;280(11):2564–71. doi: 10.1111/febs.12276 23581573

[50] Maiwald T, Hass H, Steiert B, Vanlier J, Engesser R, Raue A, et al. Driving the model to its limit: profile likelihood based model reduction. PLoS One. 2016;11(9):e0162366. doi: 10.1371/journal.pone.0162366 27588423 PMC5010240

[51] Tönsing C, Timmer J, Kreutz C. Profile likelihood-based analyses of infectious disease models. Stat Methods Med Res. 2018;27(7):1979–98. doi: 10.1177/0962280217746444 29512437

[52] Kim G-D, Liu G, Qiu D, De Leo MG, Gopaldass N, Hermes J, et al. Pools of independently cycling inositol phosphates revealed by pulse labeling with 18O-water. J Am Chem Soc. 2025;147(21):17626–41. doi: 10.1021/jacs.4c16206 40372010 PMC12123611

[53] Qiu D, Gu C, Liu G, Ritter K, Eisenbeis VB, Bittner T, et al. Capillary electrophoresis mass spectrometry identifies new isomers of inositol pyrophosphates in mammalian tissues. Chem Sci. 2022;14(3):658–67. doi: 10.1039/d2sc05147h 36741535 PMC9847636

[54] Hürlimann HC, Pinson B, Stadler-Waibel M, Zeeman SC, Freimoser FM. The SPX domain of the yeast low-affinity phosphate transporter Pho90 regulates transport activity. EMBO Rep. 2009;10(9):1003–8. doi: 10.1038/embor.2009.105 19590579 PMC2710535

[55] Wundenberg T, Mayr GW. Synthesis and biological actions of diphosphoinositol phosphates (inositol pyrophosphates), regulators of cell homeostasis. Biol Chem. 2012;393(9):979–98. doi: 10.1515/hsz-2012-0133 22944697

[56] Shears SB, Baughman BM, Gu C, Nair VS, Wang H. The significance of the 1-kinase/1-phosphatase activities of the PPIP5K family. Adv Biol Regul. 2017;63:98–106. doi: 10.1016/j.jbior.2016.10.003 27776974 PMC9351041

[57] Raia P, Lee K, Bartsch SM, Rico-Resendiz F, Portugal-Calisto D, Vadas O, et al. A small signaling domain controls PPIP5K phosphatase activity in phosphate homeostasis. Nat Commun. 2025;16(1):1753. doi: 10.1038/s41467-025-56937-0 39966396 PMC11836120

[58] Wang H, Falck JR, Hall TMT, Shears SB. Structural basis for an inositol pyrophosphate kinase surmounting phosphate crowding. Nat Chem Biol. 2011;8(1):111–6. doi: 10.1038/nchembio.733 22119861 PMC3923263

[59] Wilson MSC, Bulley SJ, Pisani F, Irvine RF, Saiardi A. A novel method for the purification of inositol phosphates from biological samples reveals that no phytate is present in human plasma or urine. Open Biol. 2015;5(3):150014. doi: 10.1098/rsob.150014 25808508 PMC4389793

