# Supplementary figures and images for: A linear pathway for inositol pyrophosphate metabolism revealed by 18O labeling and model reduction
Source: PLoS Comput Biol. 2025 Nov 10;21(11):e1013680. doi: 10.1371/journal.pcbi.1013680 (PMC12626311; doi:10.1371/journal.pcbi.1013680)

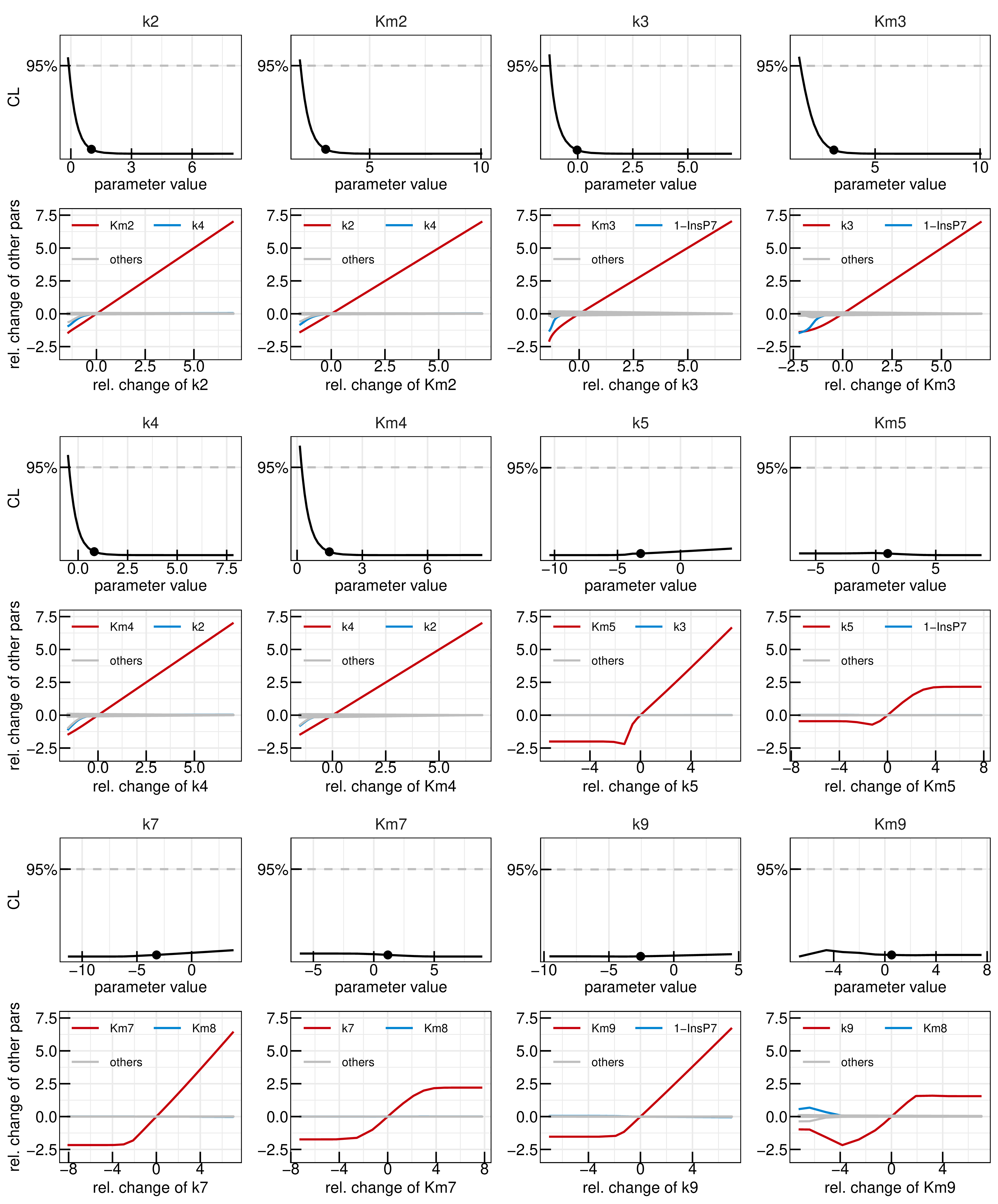

Supplement: S2 Fig — Profiles of the parameters considered for the first model reduction step in the yeast metabolic cycle. Best-fit parameter value are represented as points. Parameter values (x-axis) are displayed on log10 scale. Below each profile, the dependencies of all other parameters on the profiled parameter are plotted. Coupling strength is quantified by the relative change of a secondary parameter from its best-fit value after re-optimizing all parameters at each fixed value of the profiled parameter (x-axis). Both axes are shown on a log10 scale. The strongest dependency is highlighted in red, the next strongest in blue, and the remaining dependencies in gray. (TIFF) [file pcbi.1013680.s002.tif]

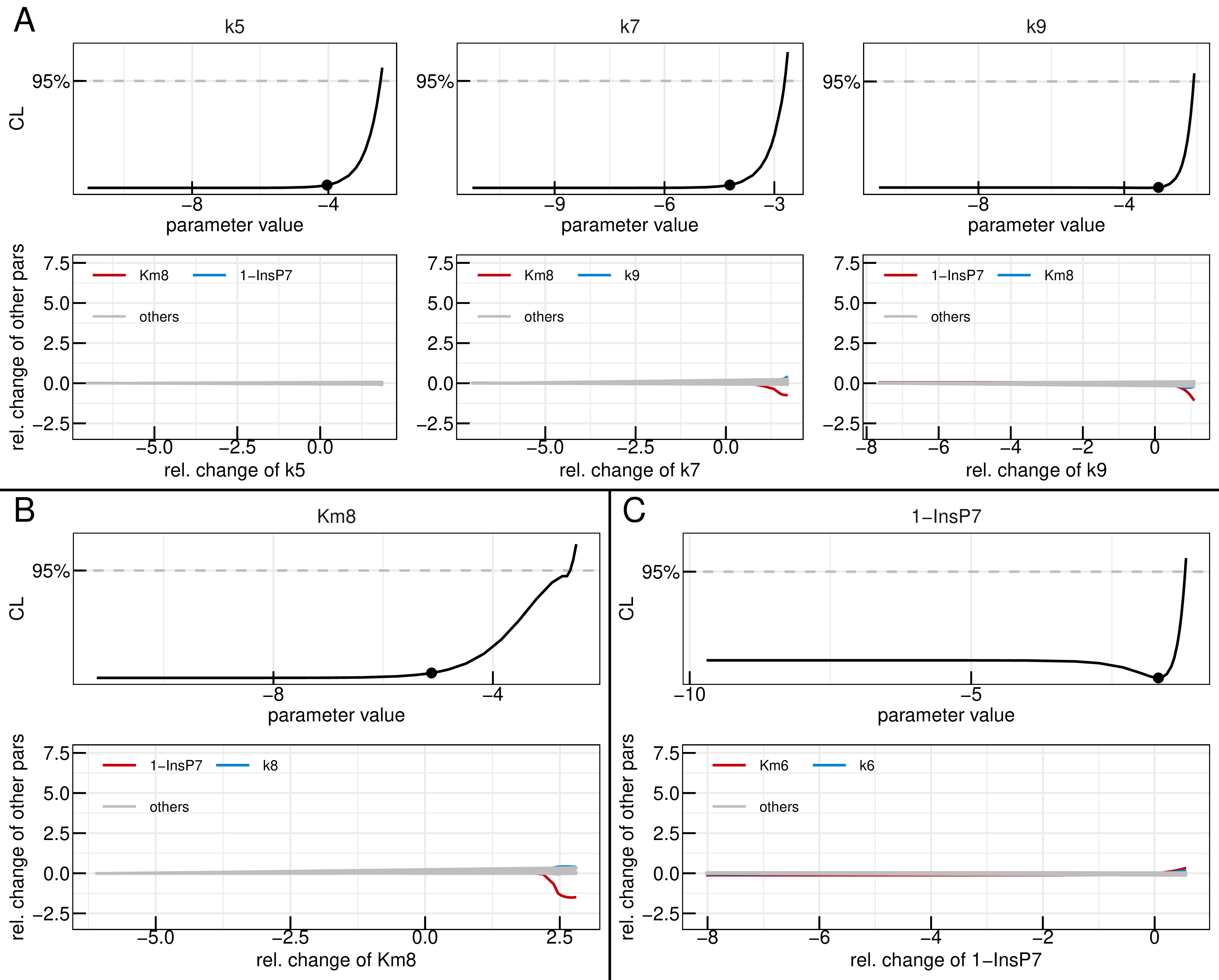

Supplement: S3 Fig — Profiles of the parameters considered for the second (A) and third (B) model reduction step in the yeast metabolic cycle. Best-fit parameter value are represented as points. Parameter values (x-axis) are displayed on log10 scale.Below each profile, the dependencies of all other parameters on the profiled parameter are plotted. Coupling strength is quantified by the relative change of a secondary parameter from its best-fit value after re-optimizing all parameters at each fixed value of the profiled parameter (x-axis). Both axes are shown on a log10 scale. The strongest dependency is highlighted in red, the next strongest in blue, and the remaining dependencies in gray. (TIFF) [file pcbi.1013680.s003.tif]

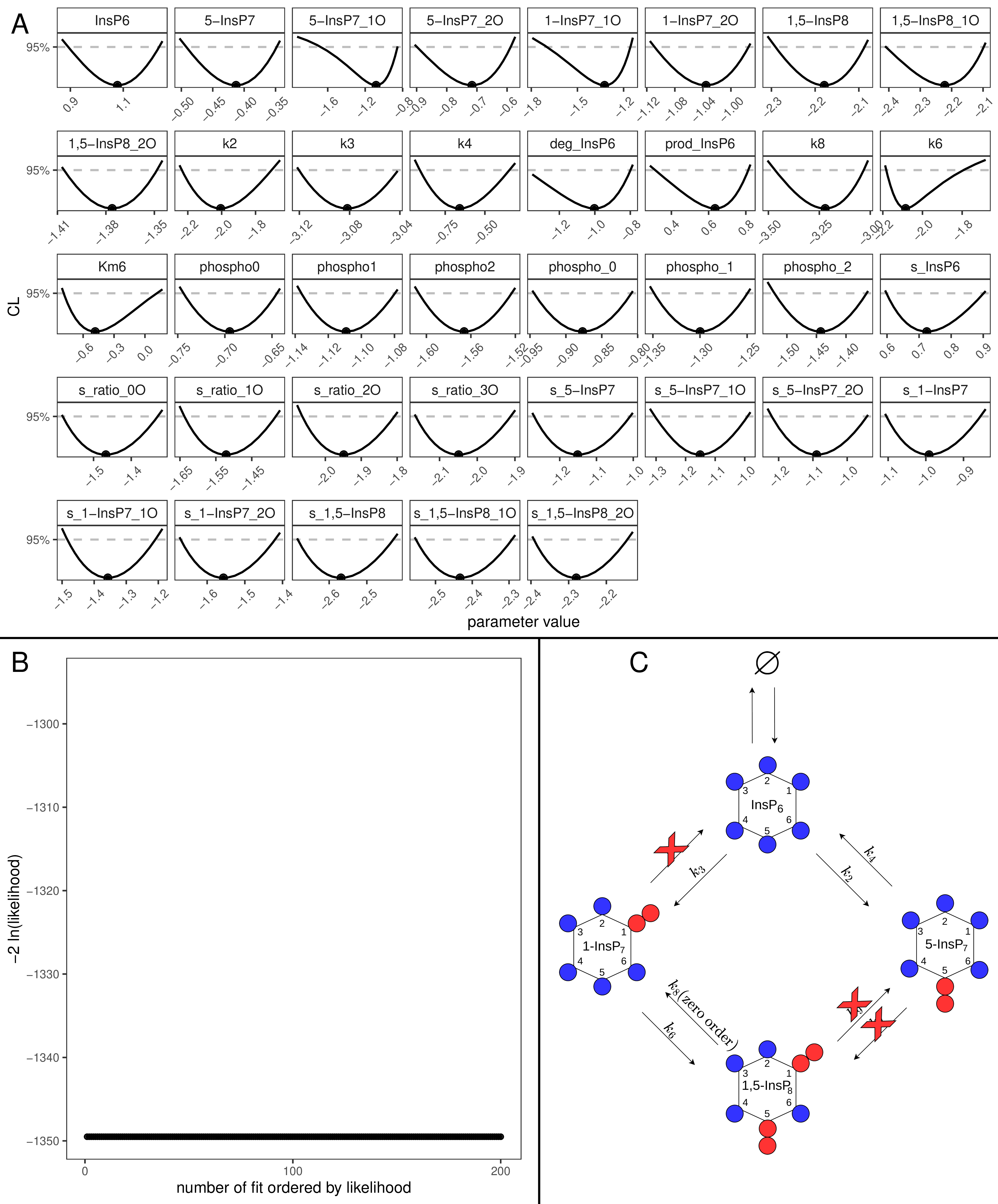

Supplement: S4 Fig — (A) Profiles of the best-fit parameters of the reduced model (line). Best-fit parameter value are represented as points. Parameter values (x-axis) are displayed on log10 scale. (B) Likelihood values of the 200 best multi-start fits, ordered by lowest likelihood value. (C) Representation of the statistically favoured transition scheme, displaying a chain-like pattern rather than a cycle, with an additional removed transition between 1-InsP7 and InsP6 compared to the normal-to-normal HCT116 data set. (TIFF) [file pcbi.1013680.s004.tif]

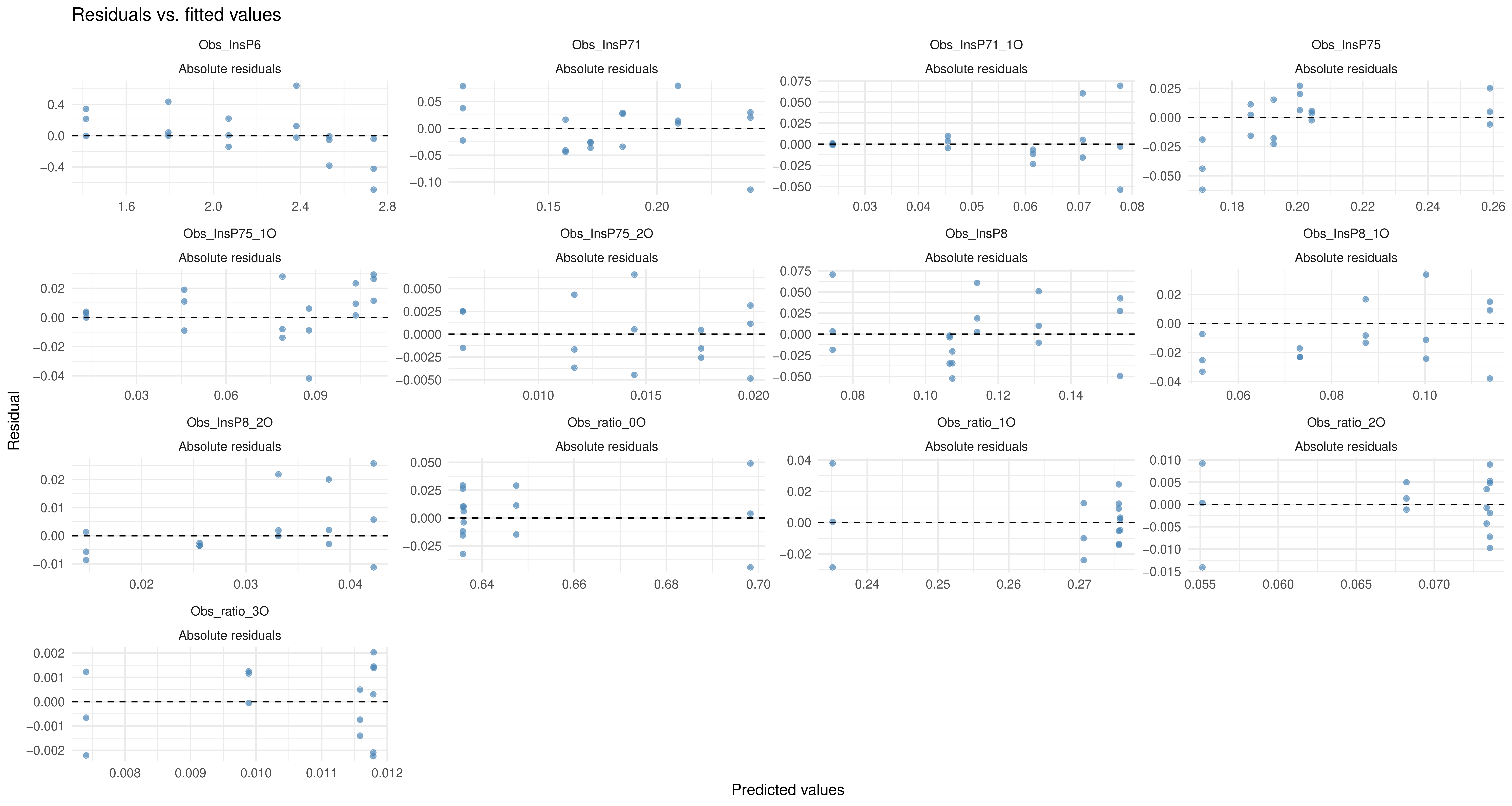

Supplement: S5 Fig — Dashed horizontal lines indicate zero residuals. A roughly constant spread of residuals across the range of predictions supports the assumption of homoscedasticity, and thus the use of an absolute error model. (TIFF) [file pcbi.1013680.s005.tif]

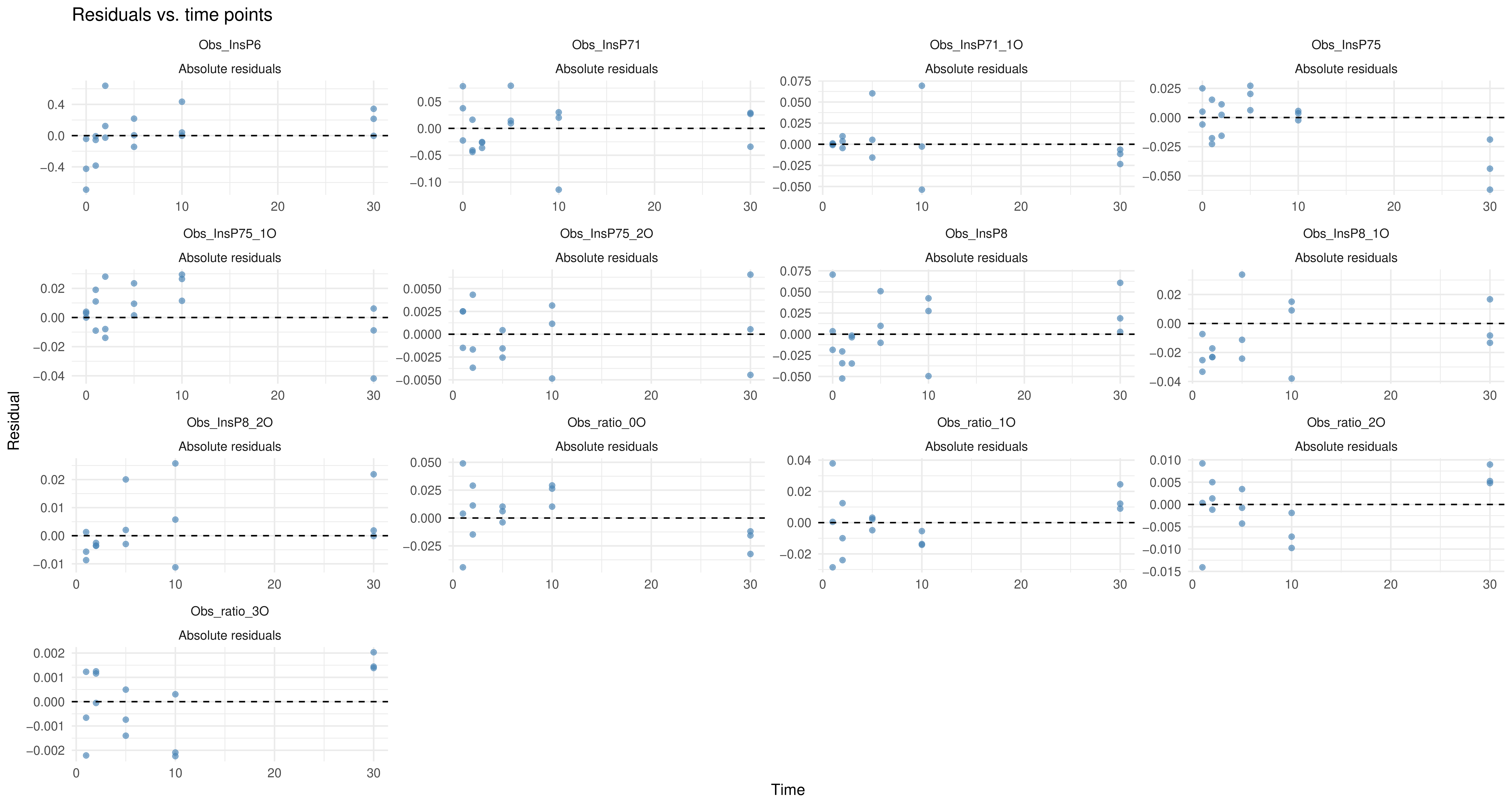

Supplement: S6 Fig — Dashed horizontal lines indicate zero residuals. A roughly constant spread of residuals across the range of time-points shows that fit is not unduly driven by certain noisy time-points. (TIFF) [file pcbi.1013680.s006.tif]
