# Supplementary material for: A linear pathway for inositol pyrophosphate metabolism revealed by 18O labeling and model reduction
Source: PLoS Comput Biol. 2025 Nov 10;21(11):e1013680. doi: 10.1371/journal.pcbi.1013680 (PMC12626311; doi:10.1371/journal.pcbi.1013680)
Supplement: Attachment — Submitted filename: Answer_letter.pdf [file pcbi.1013680.s010.pdf]

Reviewer #1, Point 1: "There is heavy reliance on mathematical modeling without experimental validation (e.g., genetic knockouts) to confirm the linear pathway. This should be more explicitly recognized, suggesting future work to validate the findings. For example, which targeted experiments could be carried out to validate the model's predictions biologically?"

Response: We now include knockout data from yeast strains (WT, *vip1*Δ, *kcs1*Δ) obtained under the same low → high Pi conditions used in our flux analysis. Importantly, while the *vip1*Δ strain shows InsPP patterns as expected, the *kcs1*Δ strain exhibits concentration changes consistent with a disruption at the transition between 1-InsP7 and InsP6 precisely as predicted by our model reduction. These results provide direct, independent support for the linear topology inferred from our ^{18}O pulse-labeling and modeling.

This new dataset is incorporated in the Results section immediately following the yeast modeling analysis (new Fig. X). Although these data were not obtained in ^{18}O-labeled medium and therefore do not provide flux estimates, they serve as a strong independent validation of the pathway topology.

In addition, we now explicitly outline future strategies to test our predictions, including (i) targeted knockouts or knockdowns in mammalian cells and (ii) isotope-labeling flux experiments in engineered yeast strains.

Reviewer #1, Point 2: Ruling out condition-specific findings. "The results seem to be based on specific phosphate conditions and may not generalize to other physiological states. Authors should discuss this possibility and how to test it."

Response: We agree that phosphate availability has a major impact on InsPP metabolism and that our results therefore reflect condition-specific flux states rather than universal behavior. In yeast, we analyzed low → high Pi transitions because this regime allows reliable detection of ^{18}O-labeled 1-InsP$_7$, which is otherwise too scarce to quantify. In HCT116 cells, both normal and low → high Pi conditions were measured, but the normal → normal regime produced the most robust and interpretable fits; for this reason, we kept these data in the main text while presenting the low → high dataset in the Supplement (Fig. S1-4).

We now explicitly state in the Discussion that flux topologies are condition-dependent: near equilibrium under constant Pi, versus synthesis-driven under Pi overplus. We emphasize that future work will be required to determine how general the linear topology is across different nutrient states and cell types.

Reviewer #1, Point 3: Limited mechanistic insights. "Authors should provide better explanation of why certain reactions dominate (e.g., enzymatic properties of IP6Ks/PPIP5Ks)."

Response: We agree that mechanistic interpretation is important. In yeast, model reduction eliminated the InsP$_6$ → 1-InsP$_7$ step, leaving InsP$_8$ synthesis via the 5-InsP$_7$ route. This is consistent with previous biochemical reports showing that Vip1/PPIP5K exhibits higher catalytic efficiency toward InsP$_7$ than InsP$_6$, suggesting that this preference also holds in vivo.

In human HCT116 cells, by contrast, model reduction eliminated the 5-InsP$_7$ → InsP$_8$ step, leaving InsP$_8$ synthesis via the 1-InsP$_7$ route. This outcome was unexpected, as it points to a flux distribution that differs from yeast and suggests species-specific differences in the relative contributions of IP6Ks and PPIP5Ks.

We have expanded the Discussion to highlight this contrast, emphasizing that the results provide mechanistic insight into how enzymatic properties shape flux distributions in vivo, and that these effects may differ between organisms.

Reviewer #1, Point 4: Model reduction. "The model reduction process is complex and inaccessible to non-specialists. It would be helpful to add a simplified explanation or visual guide for model reduction steps."

Response: We agree that a simplified overview is useful for non-specialist readers. In the revised manuscript we therefore added a schematic workflow (new Fig. Y) that illustrates the stepwise reduction procedure: (i) starting from the full model, (ii) identifying non-identifiable parameters via profile likelihoods, (iii) removing or fixing parameters associated with flat profiles, and (iv) arriving at the reduced topology. This figure is accompanied by a short explanatory paragraph in the Results section.

This visual representation complements the detailed Methods description and allows readers unfamiliar with profile likelihood–based model reduction to follow the logic of the process at a glance.

Reviewer #1, Point 5: Model assumptions. "The study assumes Michaelis–Menten kinetics, potentially oversimplifying IPP metabolism. Authors should discuss the limitations of these assumptions and the role that alternative kinetics could play (e.g., allosteric regulation)."

Response: We agree that Michaelis–Menten kinetics is a simplification and cannot capture possible phenomena such as cooperative binding, substrate inhibition, or allosteric regulation. At the same time, Michaelis–Menten kinetics is a widely used approximation in metabolic modeling and provides a pragmatic baseline in the absence of detailed in vivo rate laws.

In our framework, this formulation serves mainly as a flexible starting point: through the model–reduction procedure, the data themselves indicate whether a given reaction effectively behaves closer to mass–action, zero–order, or remains in the full Michaelis–Menten regime. We have revised the text to better emphasize both the limitations of the current approach and the need for future biochemical and kinetic studies to refine these assumptions.

Reviewer #1, Point 6: Statistical versus biological relevance. "Model reduction may overlook low-flux but biologically relevant pathways. How could this scenario be taken into account?"

Response: We agree that statistical model reduction can in principle eliminate pathways that carry only low flux but might nonetheless be biologically important, for example in signaling contexts. In the revised Discussion we now explicitly acknowledge this limitation.

At the same time, the additional knockout validation included in the revision (WT, *vip1*Δ, *kcs1*Δ) provides reassurance that the key eliminated reactions are not essential under the tested conditions. The *kcs1*Δ strain in particular shows InsPP concentrations consistent with the same disruption in the flux network predicted by model reduction, supporting the biological relevance of the reduced topology.

Nevertheless, we emphasize that our current data do not exclude the possibility that minor routes operate at low flux but may still have functional consequences. We now note that future studies with higher-sensitivity isotope tracing or targeted perturbations will be needed to address the contribution of such low-flux pathways.

Reviewer #2, Point 1: Title and framing. "The phrase 'The break in the cycle' is metaphorically appealing but scientifically imprecise. It lacks direct reference to the biological system or the specific reaction cycle under investigation. A more informative and accurate title reflecting the metabolic recharacterization would serve the scientific audience better."

Response: We agree that the original title was too metaphorical and have revised it to be more direct and descriptive. The new title is:

"A Linear Pathway for Inositol Pyrophosphate Metabolism Revealed by ^{18}O Labeling and Model Reduction."

This wording explicitly identifies the biological system (inositol pyrophosphates), the central finding (linear pathway), and the methodological approach (pulse-labeling and model reduction).

Reviewer #2, Point 2: Introduction, Lines 35–37. "The authors mention that IPPs can induce 'non-enzymatic pyrophosphorylation' of proteins such as CK2, AP3, and dynein. However, this concept is not linked to any model assumption or rate law within the paper. If non-enzymatic events are critical to IPP signaling, they should be explicitly addressed or justified as excluded in the model structure."

Response: We agree that this statement was not directly relevant to the modeling framework. To avoid confusion, we have removed the sentence on non-enzymatic pyrophosphorylation from the Introduction. This phenomenon is outside the scope of the present study, which focuses on phosphate fluxes within the metabolic pathway itself.

Reviewer #2, Point 3: Introduction, Lines 73–87. "The rationale for testing the cycle hypothesis is not clearly defined. The authors mention competing routes to 1,5-InsP$_8$ but do not articulate a falsifiable hypothesis. A clearer biological objective would strengthen the modeling aim."

Response: We agree and have revised the Introduction to state our hypothesis more explicitly. Previous proposals for 1,5-InsP$_8$ synthesis were based on in vitro assays, where the physiological contribution of alternative routes remained uncertain. Our objective was therefore to test, under in vivo conditions, whether fluxes are consistent with a cyclic topology that requires both the 1-InsP$_7$ and 5-InsP$_7$ branches, or whether the data support a simpler linear pathway.

We now frame this as a falsifiable hypothesis: *If both the 1-InsP$_7$ and 5-InsP$_7$ branches are required to reproduce the labeling dynamics, the cyclic model should be retained. However, if one branch can be consistently eliminated during model reduction without loss of fit, then a linear topology provides the most parsimonious explanation consistent with the data.*

Reviewer #2, Point 4: Results, Lines 100–113. "While the manuscript employs pulse-labeling data for flux inference, there is no description of how many biological or technical replicates were used per time point, though data suggests 3 samples. Given the known variability in mass spectrometry-based isotopologue quantification, this is a serious omission. The possibility that noisy signal from low-abundance species (e.g., 1-InsP$_7$, 5-InsP$_7$) influenced model reduction should be explicitly acknowledged."

Response: All labeling experiments were performed with three biological replicates per condition and time point, and this information has now been added to the Methods section.

We also acknowledge that variability is higher for low-abundance species such as 1-InsP$_7$ at early time points. This limitation is inherent to MS-based isotopologue quantification and we now state it explicitly in the revised text. Importantly, residual analysis indicates that the fits do not suffer from systematic bias due to this variability, and the same pathway elimination predicted by model reduction is independently supported by the *kcs1*Δ knockout data. Together, these points give us confidence that the main conclusions are robust despite noise in low-abundance measurements.

Reviewer #2, Point 5: Results, Lines 115–188 (Yeast model). "Technically, the dynamic profile fits could be biased to the fitting model which miss out on some experimental data at some timepoints and as such reduce several reactions to zero (e.g., k3, k5) or remove initial values (e.g., singly labeled 5-InsP$_7$). However, this may reflect a lack of experimental signal rather than true biological inactivity. Another example, early time points for 1-InsP$_7$ may carry high coefficient of variation, which flattens profile likelihoods and artificially promotes parameter elimination. No residual plots or CV-aware model fitting diagnostics are provided."

Response: We agree that limited signal in low-abundance species makes it difficult to fully disentangle biological inactivity from measurement variability. We now explicitly acknowledge this as a limitation in the Discussion. To address the reviewer's concern, we added residuals-versus-time plots for the yeast dataset (Supplementary Fig. S6). These show that residuals

scatter around zero without systematic bias across the time course, indicating that the fits are not unduly driven by individual noisy points.

We recognize, however, that modeling alone cannot completely rule out minor flux contributions that fall below the detection threshold. This is why independent validation is essential. In our revision, the *kcs1*Δ and *vip1*Δ knockout data provide such validation, showing the same pathway break predicted by model reduction. Taken together, these results give us confidence that the linear pathway reflects underlying biology rather than being an artifact of noise.

Reviewer #2, Point 6: Results, Lines 189–226 (HCT116 model). "The model reduction pathway differs under two phosphate conditions, yet this is not discussed as a limitation or potential modeling artifact. The claim that linear topology is a universal conclusion is overstated given these divergent results."

Response: We agree that phosphate availability influences the details of the model outcome, and we have clarified this in the revised text. Under normal Pi conditions, InsPP pools are near equilibrium, whereas under low → high Pi transitions, InsPP start to accumulate. These regimes naturally give rise to different effective flux distributions.

At the same time, we note that in both conditions the break in the cycle occurs at the same link, leading in each case to the same linear topology. This consistency argues against the result being a modeling artifact and instead supports its biological relevance. We have revised the Discussion to make clear that (i) flux distributions are condition-dependent, but (ii) the linear pathway and the specific point of cycle disruption are robust features observed across both regimes.

Reviewer #2, Point 7: Figures 2B, 5B, 6B, 9B (Fitness Landscape). "The authors present waterfall plots of the top 50 multi-start fits to claim global optimality. However, with 8–12 free parameters and only 200 multi-starts, this does not satisfy the law of large numbers. A global optimum in nonlinear space typically requires ¿10,000 samples or complementary diagnostics (e.g., likelihood landscapes, posterior sampling). The number of convergence basins is not quantified."

Response: We agree that sufficient sampling is important to demonstrate robustness. In the revised analysis we increased the number of multi-start optimizations from 200 to 1000 for representative models.

As expected, in the full (unreduced) model only a fraction of runs (∼10%) converged to the global optimum, with the remainder becoming trapped in higher local minima. After model reduction, however, the vast majority of runs converged to the same lowest-likelihood solution, and the waterfall plots (updated Figs. 2B, 5B, 6B, 9B) clearly show the stabilization of the optimum.

To complement this, we now include parameter distribution plots from the final reduction step (Supplementary Fig. S7-9). These show that independent runs converge tightly to the same parameter set. In addition, the reduced models exhibit fully identifiable parameter profiles. Together, these diagnostics demonstrate that the reduced models are robustly determined within our framework.

Reviewer #2, Point 8: Materials and Methods, Lines 297–312. "The error model is described as absolute but is not validated against experimental data. Given variable intensity ranges among isotopologues, a heteroscedastic error model may be more appropriate. Residual distributions or standard deviation bands should be shown to justify the error structure."

Response: We agree that the error model should be explicitly validated. In the revised manuscript we provide residuals-versus-prediction plots for the yeast dataset (Supplementary Fig. S5). These show no systematic fanning-out of residuals at higher intensities, indicating that the absolute error assumption is a reasonable approximation for our data.

In addition, standard deviation bands are already shown in the main figures as grey shaded areas, and these confirm that the observed variance is captured by the chosen error model. We have clarified this point in the Methods.

Together, the residual analysis and error bands support that the absolute error model is adequate within our framework, while we acknowledge that alternative error structures could be explored in future work.

Reviewer #2, Point 9: Mathematical Modeling, Lines 301–309. "The transitions from Michaelis-Menten to mass-action or zero-order are mathematically correct but biologically nontrivial. The assumption that $K_M \gg S$ is not supported by data on actual intracellular concentrations. The authors should provide substrate concentration ranges or simulated bounds."

Response: We agree that quantitative data on intracellular K_M values for the different enzymes would be valuable for evaluating the validity of the Michaelis–Menten approximation in this system. However, *in vivo* measurements of K_M values for the respective enzymes are currently not available. In Wundenberg, T. and Mayr, G. (2012), *in vitro* K_M values for PPIP5K/Vip1 (0.12μM) and IP6K/Kcs1 (0.9-8.4 μM) are reported, with the note that *in vivo* K_M values are expected to possibly be orders of magnitudes higher.

In our modeling framework, the choice between Michaelis–Menten, mass-action, or zero-order kinetics is not imposed a priori but emerges through model reduction, where the effective rate law is determined by the data. This is a standard and widely used approximation strategy in mathematical modeling when detailed biochemical parameters are unknown.

We have revised the Discussion to emphasize this limitation and to note explicitly that while the Michaelis–Menten formulation is a pragmatic approximation, future work integrating quantitative enzyme kinetic data will be important for refining the models.

Reviewer #2, Point 10: Optimization Protocol, Lines 325–338. "While multi-start optimization and log-space transformation are best practices, no convergence criteria are reported (e.g., gradient norm, tolerance thresholds). No variability statistics are shown across 200 runs, and there's no visual check of multimodality or parameter identifiability clusters."

Response: We agree that it is important to document optimization criteria. In the revised Methods we now specify the convergence tolerances used for the deterministic optimization (tolerance = $1 \cdot 10^{-10}$).

Regarding variability across runs, the expanded multi-start analysis (1000 runs) and updated waterfall plots (Figs. 2B, 5B, 6B, 9B) demonstrate that the reduced models consistently converge to the same optimum. We also provide parameter distribution plots for the final reduction step (Supplementary Fig. S7-9), which show that independent runs yield tightly overlapping parameter sets.

Finally, the profile likelihood analysis establishes parameter identifiability for each parameter in the reduced models. Taken together, the distribution plots and identifiable profiles provide evidence equivalent to a multimodality analysis within our framework.

Reviewer #2, Point 11: Overall. "The structural model may be underdetermined by the available data. Excessive parameter reduction based on profile flatness could eliminate biologically relevant fluxes. The conclusions about pathway linearity may be an artifact of model simplification and insufficient experimental resolution."

Response: We agree that model reduction carries the risk of discarding low-flux reactions that may nonetheless be biologically relevant, and we now state this limitation explicitly in the Discussion.

At the same time, we emphasize that the key outcome of a linear pathway is not based on statistical reduction alone. The independent knockout validation (*kcs1*Δ, *vip1*Δ) shows the

same disruption in the flux network as predicted by the model, providing biological evidence that supports the reduced topology.

Thus, while we acknowledge that some fluxes may remain below the resolution of our current data, the convergence of model reduction and experimental validation gives us confidence that a linear pathway best describes InsPP metabolism under the studied conditions.

Reviewer #2, Minor Critique 1: Figures 3 and 7. "While profile dependencies are well-illustrated, the red/blue/gray color scheme for parameter dependencies needs clearer legends. Parameter coupling should be quantified, not just visualized."

Response: We agree that the legends needed clarification. In the revised figures we now state explicitly that coupling strength is quantified by the relative change of the secondary parameters (y-axis) along the profile of the parameter under investigation (x-axis). Thus, the degree of coupling can be read directly from the plots, with color coding used only to rank the strongest dependencies.

To make this point clearer, we also added a note in the Methods explaining how coupling is quantified in the profile likelihood framework.

Reviewer #2, Minor Critique 2: Discussion, Lines 233–295. "The authors do not critically reflect on the consequences of excluding cyclic routes. Is this behavior conserved across eukaryotes? Could these pathways be condition-specific rather than universally dispensable?"

Response: We would like to clarify that cyclic routes were not excluded a priori. All initial models contained both branches of the cycle, and model reduction was applied to these full structures. The finding that one branch could be removed without loss of fit reflects the information content of the data rather than a modeling assumption. We have revised the Discussion to make this point explicit.

We now also address the implications more directly. The precise break point differs between yeast and human cells, but in human cells it is consistent across phosphate conditions. This suggests that linearization is a robust feature of InsPP metabolism, although its exact implementation may vary across species. At the same time, we acknowledge that cyclic fluxes may become relevant under physiological or stress conditions that were not tested here, and we highlight this as an important direction for future work.

Reviewer #2, Minor Critique 3: Materials and Methods, Line 311. "Typo: 'netto reactions' should be corrected to 'net reactions.' "

Response: We thank the reviewer for pointing out this oversight. The term has been corrected to "net reactions" in the revised manuscript.

Reviewer #2, Issue of Grammar, Spelling, Proofreading, etc. "The writing is clear and mostly free of grammatical issues. However, several terms ('disentangled', 'zero order', 'profile likelihood') are used without definition. The manuscript would benefit from a glossary or expanded introduction to modeling terminology. There are also a few other spots that need clarity... (e.g. line 143, Expect or except?). A thorough edit is required to ensure clarity and readability."

Response: We appreciate these careful observations. We have also carefully re-checked the manuscript for grammatical errors and typographical issues. Furthermore, we revised several passages for clarity to ensure the manuscript is accessible to a broad readership.
